# A metabolomic platform to identify and quantify polyphenols in coffee and related species using liquid chromatography mass spectrometry

**DOI:** 10.3389/fpls.2022.1057645

**Published:** 2023-01-06

**Authors:** Fernanda R. Castro-Moretti, Jean-Christophe Cocuron, Humberto Castillo-Gonzalez, Efrain Escudero-Leyva, Priscila Chaverri, Oliveiro Guerreiro-Filho, Jason C. Slot, Ana Paula Alonso

**Affiliations:** ^1^ BioDiscovery Institute and Department of Biological Sciences, University of North Texas, Denton, TX, United States; ^2^ BioAnalytical Facility, University of North Texas, Denton, TX, United States; ^3^ Department of Plant Science and Landscape Architecture, University of Maryland, College Park, MD, United States; ^4^ School of Biology and Natural Products Research Center Centro de Investigaciones en Productos Naturales (CIPRONA), University of Costa Rica, San Jose, Costa Rica; ^5^ Centro Nacional de Alta Technologia-Consejo Nacional de Rectores (CeNAT-CONARE), National Center for Biotechnological Innovations (CENIBiot), San Jose, Costa Rica; ^6^ Coffee Center Agronomic Institute, Campinas, Sao Paulo, Brazil; ^7^ Department of Plant Pathology, The Ohio State University, Columbus, OH, United States

**Keywords:** LC-MS/MS, phenolics, phytochemicals, *Rubiaceae*, secondary metabolism

## Abstract

**Introduction:**

Products of plant secondary metabolism, such as phenolic compounds, flavonoids, alkaloids, and hormones, play an important role in plant growth, development, stress resistance. The plant family *Rubiaceae* is extremely diverse and abundant in Central America and contains several economically important genera, e.g. *Coffea* and other medicinal plants. These are known for the production of bioactive polyphenols (e.g. caffeine and quinine), which have had major impacts on human society. The overall goal of this study was to develop a high-throughput workflow to identify and quantify plant polyphenols.

**Methods:**

First, a method was optimized to extract over 40 families of phytochemicals. Then, a high-throughput metabolomic platform has been developed to identify and quantify 184 polyphenols in 15 min.

**Results:**

The current metabolomics study of secondary metabolites was conducted on leaves from one commercial coffee variety and two wild species that also belong to the *Rubiaceae* family. Global profiling was performed using liquid chromatography high-resolution time-of-flight mass spectrometry. Features whose abundance was significantly different between coffee species were discriminated using statistical analysis and annotated using spectral databases. The identified features were validated by commercially available standards using our newly developed liquid chromatography tandem mass spectrometry method.

**Discussion:**

Caffeine, trigonelline and theobromine were highly abundant in coffee leaves, as expected. Interestingly, wild *Rubiaceae* leaves had a higher diversity of phytochemicals in comparison to commercial coffee: defense-related molecules, such as phenylpropanoids (e.g., cinnamic acid), the terpenoid gibberellic acid, and the monolignol sinapaldehyde were found more abundantly in wild Rubiaceae leaves.

## 1 Introduction

Plant secondary metabolites are byproducts of primary metabolism. They play important roles during plant development, reproduction and stress response (Patra et al., 2013; Ma et al., 2016; Böttger et al., 2018; Kessler and Kalske, 2018; Jain et al., 2019). Because plants are sessile organisms, they must endure environmental and biotic pressure. The production of phytochemicals is part of their response to these stresses. Besides their importance for plant adaptation, growth and development, plant secondary compounds are valuable resources for the food, pharmaceutical, and biofuel industries (Boudet, 2007; Korkina, 2007; Cragg and Newman, 2013; Chiocchio et al., 2021). Alkaloids, phenolics, and terpenoids are the three main families that comprise secondary metabolites produced by plants. They are synthesized through malonic acid, mevalonic acid, methylerythritol-phosphate, and shikimate pathways (Jain et al., 2019). Because of their vast structural/chemical diversity, low solubility, and small quantities in plant tissues, the recovery, identification and quantification of phytochemicals are particularly challenging.

Genetics studies in combination with metabolic profile are key for plant breeding and insertion of desired traits, such as specific polyphenols. Recovering lost attributes due to domestication using wild relatives in the breeding program is a promising strategy. However, it has been mostly applied to crops such as rice, wheat, barley and potatoes (McSorley and Phillips, 1992; Peleg et al., 2005; Feuillet et al., 2008; Spooner et al., 2014; Brar and Khush, 2018). Despite the limited number of studies on coffee leaf and other *Rubiaceae* metabolic content, the presence of phenolic compounds has been previously described (Souard et al., 2018; Cangeloni et al., 2022; Montis et al., 2022). Caffeine, chlorogenic acids, mangiferin and trigonelline are the main phytochemicals found in coffee leaves (Cangeloni et al., 2022). Indole alkaloids are the most common secondary metabolite class throughout *Rubiaceae* species, although other classes of alkaloids, terpenes and flavonoids have also been reported (Martins and Nunez, 2015). For example, akuamigine, vincoside, yohimbine and other indole alkaloids have been detected in the *Rubiaceae Uncaria* spp. (Laus and Teppner, 1996; Ndagijimana et al., 2013; Xie et al., 2013). Different species of *Gardenia* sp. produce iridoids, such as genipin and gardenoside, as well as flavonoids and triterpenes (Chen et al., 2009; Kunert et al., 2009; Yang et al., 2013; Wang et al., 2015). Many members the *Rubiaceae* family have been studied for their secondary metabolites with medicinal properties (Chen et al., 2009; Ahmad and Salim, 2015; Martins and Nunez, 2015). For instance, species that are used in traditional medicine from the genera *Borreria* and *Spermacoce* contain alkaloids, flavonoids, iridoids, and terpenoids (Conserva and Ferreira Júnior, 2012). Additionally, medicinal plants with known anti-inflammatory and antioxidant properties, such as species from the genera *Rytignia* and *Canthium multiflorum*, have bioactive compounds like tannins, saponins and flavonoids, coumarins and terpenoids (Chandra Kala, 2015). Therefore, it is important to develop efficient methodologies to monitor plant polyphenols, which will guide breeding programs and boost phytochemical discovery.

In order to fully grasp the diversity of phytochemicals present in leaves of coffee and other *Rubiaceae* species, i) a single extraction procedure allowing to recover the vast diversity phytochemical families is needed, ii) an untargeted metabolomics approach is required to detect unknown/new polyphenols, and iii) high-throughput targeted metabolomics method is necessary to quantify a maximum of secondary compounds within a single run. Developing a fast methodology that isolates most of the secondary metabolites present in leaves is challenging. 

Metabolomics is the ideal technique for detecting small quantities of phytochemicals (Jorge et al., 2016; Cocuron et al., 2019; Castro-moretti et al., 2020). Nuclear magnetic resonance (NMR) and mass spectrometry (MS) are the most common analytical tools for performing plant metabolomics. MS coupled with liquid or gas chromatography (LC and GC, respectively) is a preferred method due to its higher sensitivity and lesser amount of sample requirements (de Falco and Lanzotti, 2018; Liu et al., 2019; Perez de Souza et al., 2019). In this study, two MS instruments were used: a high-resolution quadrupole time-of-flight (HR-Q-TOF) for untargeted metabolomics, and a highly sensitive triple quadrupole for targeted quantification of known metabolites. On one hand, untargeted studies are designed to detect a broad range of molecules in a biological sample (Patti et al., 2012; Perez de Souza et al., 2019). It is common to use spectral libraries to attempt compound identification (Dunn et al., 2012; Perez de Souza et al., 2019; Jez et al., 2021). On the other hand, targeted metabolomics is used to quantify known metabolites using analytical standards (Patti et al., 2012; Roberts et al., 2012; Sawada and Yokota Hirai, 2013). Although complementary, these two approaches have been rarely combined (Montis et al., 2022).

Other approaches have attempted to isolate and quantify plant secondary metabolites; however, a limited number of families (one or two) and compounds (less than 40) were monitored (Sun et al., 2013; Orcic et al., 2014; Bataglion et al., 2015; Lin et al., 2015; Jaini et al., 2017; Cocuron et al., 2019; Gulcin et al., 2019; Marchetti et al., 2019; Quatrin et al., 2019). In this study, a single-extraction method was developed to recover 42 distinct families of phytochemicals. Untargeted and targeted metabolomics were combined to study the secondary metabolites present in coffee and wild *Rubiaceae* leaves. More specifically, a state-of-the-art targeted approach allowing the quantification of 184 phytochemicals was developed and was used to validate the identity of 74 compounds highlighted by the untargeted analysis. Combining both techniques and instruments along with an optimized extraction method resulted in a sensitive and thorough pipeline to detect, classify and quantify secondary metabolites in leaves of coffee and other *Rubiaceae* species. We anticipate that this thorough pipeline will boost the process of detection, classification and quantification of polyphenols in leaves of coffee and other *Rubiaceae*, and will be further applied to other plant organs and species.

## 2 Materials and methods

### 2.1 Chemicals

LC-MS-grade acetic acid, acetonitrile, methanol, DMSO, and water were ordered from Thermo Fisher Scientific (Hampton, NH). All non-labeled standards as well as trans-cinnamic acid-β,2,3,4,5,6-d6 were purchased from MilliporeSigma (Burlington, MA). N,N-dimethyltryptamine (N,N-DMT), bufotenin (5-OH-DMT) and psilocybin standards were obtained from Cayman Chemical (Ann Arbor, MI).

### 2.2 Preparation of standard stocks and working solutions

Stock solutions of dihydrokaempferol, dihydroquercetin, mitragynine, luteolin, luteolin-7-O-glucoside, naringenin, phloretin, piceid, prunetin, pterostilbene, orientin, quercetin, quercitrin, reserpine, rhamnazin, rhamnetin, schaftoside, spiraeoside, swertiajaponin, swertisin, tectochrysin, tricin, vicenin 2 and 3, vincosamide and yohimbine were prepared at 1,000 µM. Acacetin-7-O-rutinoside, afzelin, apigenin-7-glucuronide, calycosin, corynanthine, harmane, hordenine, ipriflavone, tomatidine, xanthohumol, sophoricoside, rauwolscine, idaein, keracyanin and neobavaisoflavone were prepared at 100 µM. All other stock solutions were prepared at 10,000 µM, using methanol or DMSO as solvents. Working solutions were prepared to final concentrations of 100, 50, 10 and 1 µM in methanol/water (40:60; v/v).

### 2.3 Leaf collection

Untargeted and targeted metabolomic analyses were performed using commercial coffee leaves (*Coffea arabica* cv. Obatã IAC 1669-20 - CC) that were collected in San José, Costa Rica, at the Coopetarrazú plantation. Three mature leaves were harvested from four trees and kept on ice during transportation to the laboratory, where they were flash-frozen in liquid nitrogen, and lyophilized until dryness. Wild *Rubiaceae* leaves were collected from the two species *Isertia hankeana* and *Simira maxonii* (WR1 and WR2, respectively) in a private rainforest of the Golfito (Puntarenas) region of Costa Rica. Three leaves were collected from each tree, one tree per species and kept in ice during their transportation to the laboratory. Then they were flash-frozen in liquid nitrogen and lyophilized (Labconco Freezone, South Kansas City, KS) until dryness. To optimize the extraction and LC-MS/MS method, mature leaves from the wild coffee species *Coffea liberica* var. *dewevrei* and *Coffea salvatrix* were used from plants grown in field conditions, collected at the Agronomic Institute in Campinas (São Paulo, Brazil). Four leaves were collected from each tree, two trees per species, kept in dry ice during harvest and transportation to the laboratory and then they were frozen in liquid nitrogen prior lyophilization until dryness. All collected leaves were lyophilized using a freeze dryer Labconco Freezone 12 plus (South Kansas City, KS). Dried leaves were ground into a fine powder using 15 mL plastic jars with four 20 mm metal beads in a tissue homogenizer Geno/Grinder 2010 from Spex (Metuchen, NJ) for two rounds of 30 sec at 1,750 rpm. Wild *Rubiaceae* leaves were collected from the same tree, grown in very different environmental conditions in comparison to CC; to mitigate variations due to leaf maturity level, light exposure, etc., and to focus on polyphenol differences across species, the ground powder was pooled, homogenized and divided into five pseudoreplicates per species.

### 2.4 Intracellular secondary metabolite extraction

The extraction of leaf secondary metabolites was performed after grinding and weighting 10 mg of powdered leaf material. Metabolites were extracted by adding 10 µL of 1 mM trans-cinnamic acid-β,2,3,4,5,6-d6 and 490 µL of 100% methanol followed by grinding with one 5 mm metal bead at 30 Hz for 5 min using a mixer mill MM400 from Retsch (Haan, Germany). Then, the extracts were sonicated at 35-40°C for 20 min and centrifuged at 9,600 *g* for 5 min at room temperature. The supernatants were transferred to 1.5 mL microcentrifuge tubes. The remaining pellets were resuspended in 500 µL of methanol/water (30:70, v/v), sonicated for 20 min at 35-40 °C, and spun down under the same conditions as mentioned before. The supernatants were combined to the first ones, and then, 500 µL of extracts were filtered through 3 kDa Amicon filtering devices (MilliporeSigma, Burlington, MA) at 14,000 *g* for 60 min at room temperature. The resulting eluates were stored at -20°C until LC-MS/MS analysis.

### 2.5 Untargeted metabolomics

The analysis of the metabolites was carried out using an Exion ultra high-performance liquid chromatography system coupled with a high-resolution mass spectrometer TripleTOF6600+ from AB Sciex (Framingham, MA).

#### 2.5.1 HPLC conditions

The compounds were separated using a C18 Symmetry column (75 x 4.6 mm, 3.5 µm) with a Symmetry C18 pre-column (20 x 3.9 mm; 5µm) from Waters (Milford, MA) as previously described (Cocuron et al., 2019). The temperatures of the column compartment and the autosampler were kept at 30 °C and 15 °C, respectively. The analytes were eluted using a gradient of 0.1% (v/v) acetic acid in acetonitrile (Solvent A) and 0.1% (v/v) acetic acid in water (Solvent B) under a flow rate of 0.8 mL/min. The following gradient was applied: 0-1.0 min, 98% B; 1.0-16.0 min, 98-42% B; 16.0-21.0 min, 42-20% B; 21.0-26.0 min, 20-10% B; 26.0-28.0 min, 10% B; 28.0-28.1, 10-98% B; 28.1-30.0 min, 98% B.

#### 2.5.2 High-resolution discovery using triple TOF

##### 2.5.2.1 Data-dependent acquisition

The mass spectrometer was set to scan metabolites from m/z 100-1500 amu in negative or positive mode. For the negative polarity the ion spray voltage was 4,500 V, the accumulation time was 100 msec, the declustering potential and collision energy were 60 V and 10 V, respectively. MS/MS spectra were acquired over m/z 30-1500 amu with an accumulation time of 25 msec. Parameters such as declustering potential, collision energy, collision energy spread were set to 60 V, 45 V and 15 V, respectively. The parameters for the positive mode were very similar to the ones for the negative mode except for the ion spray voltage, and the declustering potential that were 5,000 V and 35 V, respectively. The total cycling time was 0.65 sec.

The parameters for the electrospray source ionization such as curtain gas (nitrogen), nebulizing gas, heating gas, and the temperature of the source were fixed at 40 psi, 70 psi, 70 psi, and 650 °C, respectively. The source conditions were the same for the negative and positive polarities. An atmospheric-pressure chemical ionization (APCI) negative or positive calibration solution was delivered by a calibrant delivery system every 5 samples to correct for any mass drift that may occur during the run. MS spectra were acquired using Analyst TF 1.8.1 software (AB Sciex, Framingham, MA). It is important to note that the data-dependent acquisition (DDA) was run in negative and positive modes, on a mixture composed of CC, WR1 and WR2 extracts. The precursor ions present in this mixture were then used for the sequential window acquisition of all theoretical mass spectra (SWATH-MS) scan survey.

##### 2.5.2.2 Data-independent acquisition using SWATH-MS

The precursor ion data obtained from the DDA (negative and positive ionizations) were used to generate short overlapping precursor ion windows which are the core of the SWATH-MS mode. Briefly, all precursors ions from the DDA mode (negative or positive polarity) as well as their intensities were exported to an excel file in which a total of 20 variable SWATH-MS windows were created with one amu overlapping mass. These SWATH-MS windows for the negative or positive polarity were saved as a “.txt” file, and uploaded to Analyst to build the mass spectrometry part of the LC-HR-MS/MS acquisition method. It is important to note that the source, MS scan and MS/MS scan parameters for the SWATH-MS mode were the same than the ones used for the DDA scan survey.

For the sequence injection, the total 15 biological samples were placed randomly in the autosampler. Two quality controls (QC) consisting of an equal mixture of each leaf extract, and two blanks containing the internal standard, trans-cinnamic acid-β,2,3,4,5,6-d6 in methanol: water (40:60, v/v), were included; one was injected at the beginning of the sequence, and the other at the end.

#### 2.5.3 Data processing

A non-targeted screening MQ4 workflow was designed using the software SciexOS v.1.6.1 (Sciex, Framingham, MA) with the Smart Confirmation Search algorithm. Results were sorted by purity, 0.02 Da as precursor mass tolerance, and 0.4 Da as fragment mass tolerance. The intensity threshold was set to 0.05 and minimal purity to 10%. All extract samples, blanks with internal standard, and QCs were considered for constructing the processing method. Quality of the processed data was assessed as follows: i) the difference in the area of the internal standard between the sample extracts and the blanks was less than 5%; and ii) a Principal Component Analysis (PCA) including the QCs was performed for the metabolites monitored in negative and positive modes and showed clustering of the two QCs (**Supplemental Figure 1**), which is indicative of a low variance. Then, data obtained from untargeted metabolomics were analyzed using the software XCMS (Tautenhahn et al., 2012) with the following parameters: method UPL/UHD Q-TOF matchedFilter; ppm error of 15; minimum peak width of 5; maximum peak width of 20; signal/noise threshold of 6; mzdiff of 0.01; integration method 1; prefilter peaks 3; noise filter 0; and retention time correction method obiwarp. Once peak picking, alignment and integration was performed, a table with mass to charge ratio, retention time, and normalized intensity of each feature (by the intensity of the internal standard, trans-cinnamic acid-β,2,3,4,5,6-d6) was generated. This table was then used for statistical analyses (see 2.7. Statistical Analyses). Further data curation was performed: features with signal intensity lower than 1,000,000.00 count per second were excluded, as well as the features not present on all replicates. Then, the feature with the highest intensity was selected for each peak group. Features from positive and negative ionization were merged and, when the same feature was present in both modes, the one with the highest intensity was selected. Metabolite identification was performed using the software SciexOS v.1.6.1 (Sciex, Framingham, MA) with spectral libraries from the National Institute of Standards and Technology (NIST, Gaithersburg, MA) and a homemade library.

### 2.6 Targeted metabolomics using LC-MS/MS scheduled multiple reaction monitoring

#### 2.6.1 LC-MS/MS conditions

The detection and quantification of phytochemicals was performed as previously described by (Cocuron et al., 2019) with some slight modifications concerning the LC gradient and the use of scheduled multiple reaction monitoring (sMRM).

The 184 phytochemicals considered in this study were optimized one by one by direct infusion after having been diluted to 1 µM with acetonitrile/water solution (50:50; v/v) containing 0.1% of acetic acid as an additive. The flow for the direct infusion was set to 10 µL/min, and parameters such as declustering potential (DP), collision energy (CE), cell exit potential (CXP) were determined for the five most abundant product ions derived from each precursor ion (see **Table 1**). The compound optimization was done automatically for the negative and positive polarities using Analyst 1.7 software (AB Sciex, Framingham, MA). The source optimization for the electrospray ionization was conducted using different values for the curtain gas (25, 30, 35, 40 V), the nebulizer gas (GS1; 40, 45, 50, 55, 60, 65 V), the heating gas (GS2; 40, 45, 50, 55, 60, 65 V), the collision activated dissociation (CAD; low, medium, high), the temperature (300, 350, 400, 450, 500, 550, 600, 650°C), and the ionspray voltage (IS; 3000, 3500, 4000, 4500, 5000 V).

**Table 1:** Compound-dependent parameters for scheduled MRM scan survey, per metabolite and their chemical class (family): retention time (RT) in minutes, precursor mass (Q1) and product mass (Q3), declustering potential (DP), collision energy (CE) and collision cell exit potential (CXP) in volts.

Family	Metabolite	RT	Q1	Q3	DP	CE	CXP
alkaloid	Corynanthine	4.1	355.3	144.0	113	39	16
alkaloid	Dihydrocinchonine	4.2	297.0	279.0	40	31	14
alkaloid	Harmane	3.6	183.0	115.0	50	45	12
alkaloid	Hordenine	1.1	166.0	120.9	24	21	14
alkaloid	Mitragynine	4.9	399.0	174.0	75	41	20
alkaloid	Seneciphylline	3.4	334.0	120.0	105	35	14
alkaloid	Tomatidine	5.6	416.0	161.0	91	49	18
amino acid derivative	5-Hydroxy-tryptophan	3.1	221.0	204.0	20	13	12
amino acid derivative	Tyramine	0.9	138.0	120.9	9	13	14
anthocyanidin	Apigeninidin	4.4	255.0	170.9	127	43	18
anthocyanin	Idaein	3.7	449.1	287.0	60	29	14
anthocyanin glycoside	Keracyanin	3.6	595.2	286.9	75	39	14
benzodioxol	Piperonyloyl	6.4	184.8	142.8	50	13	16
chalcone	Xanthohumol	9.6	355.0	299.0	27	15	16
cinnamaldehyde	p-Coumaraldehyde	5.9	147.1	119.0	-50	-24	-13
cinnamate ester	3,4-Di-O-caffeoylquinic acid	6.6	515.0	353.0	-80	-28	-19
cinnamate ester	3-Caffeoylquinic acid	4.7	353.0	134.0	-25	-62	-15
cinnamate ester	4,5-Di-O-caffeoylquinic acid	6.7	515.0	353.0	-80	-28	-19
cinnamate ester	4-Caffeoylquinic acid	5.3	353.0	173.0	-45	-20	-11
cinnamate ester	5-Caffeoylquinic acid	4.8	353.0	93.0	-25	-56	-11
coumarin	6-Methylcoumarin	7.7	161.0	105.0	80	29	12
coumarin	7,8-Dihydroxy-4-methylcoumarin	5.4	191.0	119.0	-65	-26	-11
coumarin	Scopoletin	5.5	192.9	133.0	100	29	14
coumarin derivative	Esculetin	4.7	178.9	123.0	80	31	14
coumarin derivative	Mellein	8.2	179.0	160.8	11	17	18
cyclic ketone	Isophorone	7.5	138.9	68.9	184	21	8
cyclohexenecarboxylic acid	p-Coumaroyl-shikimate	5.3	321.1	147.0	115	15	10
dihydrochalcone	Phloretin	6.9	274.9	106.9	50	21	12
diterpenoid	Gibberellic acid	5.4	345.0	239.0	-70	-20	-13
diterpenoid	Ginkgolide A	6.6	409.1	345.0	80	27	16
flavanol	Catechin	4.3	291.0	139.0	50	20	16
flavanol	Epicatechin	4.6	291.0	139.0	50	20	16
flavanol	Epigallocatechin	4.1	307.0	138.9	13	19	16
flavanol	Gallocatechin	3.7	307.1	138.9	19	19	16
flavanone	Eriodictyol	6.5	287.0	151.0	-30	-20	-7
flavanone	Hesperetin/Homoeriodictyol	7.3	303.0	177.0	85	25	10
flavanone	Isosakuranetin	8.4	287.0	153.0	80	29	18
flavanone	Naringenin	7.1	272.9	153.0	50	31	16
flavanone	Sakuranetin	8.4	287.0	167.0	90	29	20
flavanone-C-glycoside	Swertiajaponin	4.6	463.1	445.1	60	17	22
flavanone O-glycoside	Naringin	5.3	579.1	271.0	-150	-44	-13
flavanone O-glycoside	Naringenin-7-O-glucoside	5.5	435.1	273.0	70	19	14
flavanonol	Dihydrokaempferol	6.1	287.0	125.0	-60	-28	-5
flavanonol	Dihydroquercetin	5.5	303.0	285.0	-50	-16	-13
flavone	3-Deoxyrobinetin	5.1	284.9	149.0	-110	-36	-9
flavone	Apigenin	7.0	269.0	117.0	-90	-42	-13
flavone	Apigenin-7-glucuronide	6.8	447.1	271.1	120	29	14
flavone	Baicalein	7.3	270.9	122.9	150	43	14
flavone	Chrysin	8.3	253.0	143.0	-110	-36	-9
flavone	Chrysoeriol	7.0	301.0	286.0	80	37	32
flavone	Diosmetin	7.1	300.9	286.0	80	35	32
flavone	Eupatorin	7.9	344.9	283.9	100	41	30
flavone	Flavopiridol	4.8	402.0	341.0	75	33	16
flavone	Genkwanin	8.5	284.8	242.0	80	43	26
flavone	Maysin	5.5	577.0	431.0	50	19	24
flavone	Myricetin	5.8	317.0	151.0	-70	-32	-15
flavone	Scutellarein	6.0	286.9	123.0	110	45	14
flavone	Tectochrysin	10.1	268.9	226.0	80	43	26
flavone	Tricin	7.0	331.0	315.0	100	41	34
flavone C-glycoside	Isoorientin	4.6	449.0	299.0	50	39	14
flavone C-glycoside	Swertisin	5.0	447.2	297.0	60	35	14
flavone-C-glycoside	Isoschaftoside	4.5	565.1	427.1	100	29	20
flavone-C-glycoside	Isovitexin/Vitexin	4.9	433.0	283.0	80	35	14
flavone-C-glycoside	Orientin	4.7	449.0	329.0	120	39	16
flavone-C-glycoside	Rhamnosylisoorientin	4.4	593.0	298.0	-150	-58	-13
flavone-C-glycoside	Vicenin 2	4.2	595.1	577.1	100	21	28
flavone-C-glycoside	Vicenin 3/Schaftoside	4.5	565.1	547.2	80	19	26
flavone-O-glycoside	Acacetin-7-O-rutinoside	5.8	593.1	447.1	60	25	22
flavone-O-glycoside	Apigenin-7-O-glucoside	5.3	432.9	271.0	60	25	14
flavone-O-glycoside	Benzoic acid	7.4	120.9	77.0	-20	-16	-9
flavone-O-glycoside	Luteolin-7-O-glucuronide	6.5	463.0	287.0	130	29	14
flavone-O-glycoside	Myricetin-3-O-Rhamnoside	5.0	465.0	319.0	30	15	16
flavone-O-glycoside	Neodiosmin	5.3	607.0	299.0	-150	-40	-15
flavonol	Fisetin	5.9	286.9	137.0	120	43	16
flavonol	Gossypetin	5.8	319.0	169.1	160	43	18
flavonol	Isorhamnetin	7.2	315.0	300.0	-80	-28	-15
flavonol	Kaempferide	8.5	300.9	229.0	120	53	24
flavonol	Kaempferol	7.1	286.9	153.0	120	43	18
flavonol	Luteolin	6.4	286.9	153.0	120	43	18
flavonol	Morin/Tricetin	6.3	302.9	152.9	140	39	18
flavonol	Quercetin	6.5	301.0	151.0	-80	-28	-15
flavonol	Quercitrin	5.4	449.0	303.0	30	15	16
flavonol	Rhamnazin	8.6	331.0	316.0	140	35	36
flavonol	Rhamnetin	7.8	315.0	165.0	-60	-28	-9
flavonol	Tamarixetin	7.2	316.9	302.0	120	35	14
flavonol-O-glucuronide	Miquelianin	6.7	479.0	303.0	60	21	16
flavonol-O-glycoside	Isoquercetin	5.1	465.1	303.0	30	17	16
flavonol-O-glycoside	Isorhamnetin-3-O-glucoside	5.3	479.1	317.0	30	17	16
flavonol-O-glycoside	Kaempferitrin	4.8	579.1	433.1	50	17	20
flavonol-O-glycoside	Kaempferol-3-O-glucoside	5.4	449.0	287.0	40	17	16
flavonol-O-glycoside	Kaempferol-3-O-glucuronide	6.4	463.0	287.0	50	21	14
flavonol-O-glycoside	Kaempferol-3-O-rutinoside	5.1	595.1	287.1	50	27	14
flavonol-O-glycoside	Kaempferol-7-O-Neohesperidoside	5.1	595.1	287.0	120	31	14
flavonol-O-glycoside	Luteolin-7,3’-Di-O-glucoside/Kaempferol-3-O-sophoroside	4.6	611.1	449.1	150	31	22
flavonol-O-glycoside	Luteolin-7-O-glucoside/Kaempferol-7-O-glucoside	5.4	449.0	287.0	91	27	14
flavonol-O-glycoside	Quercetin-3,4’-O-diglucoside	4.5	627.0	465.0	60	19	24
flavonol-O-glycoside	Quercetin-3-O-galactoside	5.0	465.0	303.0	40	17	16
flavonol-O-glycoside	Rutin	4.8	611.2	303.0	30	29	16
flavonol-O-glycoside	Spiraeoside	5.5	465.0	303.0	120	29	16
flavonol-glycoside	Afzelin	5.7	431.1	285.0	-100	-28	-15
glycosylated hydroquinone	Arbutin	2.9	271.0	161.0	-60	-10	-9
hydroxycinnamic acid	Cynarin	5.4	515.0	190.9	-54	-40	-11
hydroxycinnamic acid	Ferulic acid	5.5	192.9	134.0	-25	-20	-9
hydroxycinnamic acid	p-coumaric acid	5.4	162.9	119.0	-25	-18	-15
hydroxycinnamyl alcohol	p-Coumaryl alcohol	5.1	149.1	131.0	-25	-14	-13
hydroxy monocarboxylic acid	Caffeoyl-shikimate	4.8	337.0	163.0	120	15	10
indole alkaloid	5-Hydroxydimethyltryptamine	1.2	205.0	160.0	40	19	10
indole alkaloid	N,N-dimethyltryptamine	3.3	189.0	144.0	25	23	16
indole alkaloid	Psilocybin	3.1	285.0	205.0	50	23	10
indole alkaloid	Rauwolscine	5.4	355.0	144.0	100	39	14
indole alkaloid	Reserpine	5.5	609.0	195.0	50	47	10
indole alkaloid	Strychnine	3.5	335.0	184.0	120	49	20
indole alkaloid	Theobromine	3.5	181.0	138.0	85	23	14
indole alkaloid	Theophylline	3.9	181.0	124.0	60	25	14
indole alkaloid	Tryptamine	3.1	161.0	144.0	20	13	10
indole alkaloid	Vincosamide	6.2	499.0	337.0	60	23	18
indole alkaloid	Yohimbine	4.0	355.0	144.0	60	37	16
indole alkaloid	Paynantheine	4.9	397.0	174.0	50	37	20
isoflavone	Calycosin	6.5	285.0	269.9	77	31	28
isoflavone	Glycitein	6.3	284.9	270.1	100	35	30
isoflavone	Ipriflavone	10.2	280.8	239.0	114	27	12
isoflavone	Neobavaisoflavone	8.4	323.0	266.9	75	25	14
isoflavone	Prunetin	8.6	284.9	241.9	150	43	26
isoflavone-glycosylated	Sophoricoside	5.4	433.0	271.0	80	17	14
isoflavone-O-glycoside	Genistein-7-O-glucuronide/Baicalin	6.9	447.0	271.0	120	27	14
isoflavone-O-glycoside	Glycitin	4.7	447.0	224.8	42	59	24
lactone	Caffeic acid	6.9	178.9	135.0	-35	-20	-15
lignan	Arctigenin	7.7	371.0	83.0	-71	-24	-15
lignan	Matairesinol	7.1	357.2	82.9	-76	-26	-9
methylxanthine alkaloid	Caffeine	4.3	195.0	138.0	150	27	14
monocarboxilic acid	Cinnamic acid	7.1	148.8	103.0	20	25	12
monohydroxybenzoic acid	4-Hydroxybenzoic acid	4.6	136.9	93.0	-15	-20	-13
monohydroxybenzoic acid	Salicylic acid	9.1	136.9	93.0	-15	-20	-13
monohydroxybenzoic acid	Vanillic acid	4.8	166.9	108.0	-50	-30	-47
mycotoxin	Neosolaniol	5.0	383.2	365.1	117	13	18
mycotoxin	Roridin-L2	7.1	531.3	249.1	29	21	14
octadecanoid	Oxo-phytodienoic acid	9.6	293.0	275.0	35	15	16
O-methylated isoflavone	Brefeldin A	8.6	281.0	245.0	13	9	14
oxopurine alkaloid	1,3,7-Trimethyluric acid	4.0	209.0	194.0	-40	-18	-9
oxylipin	Jasmonic acid	7.3	209.0	59.0	-80	-16	-27
oxylipin	Methyl jasmonate	8.9	225.0	151.0	20	17	10
phenol	Gingerol	8.4	292.8	99.1	-60	-16	-11
phenolic	Rosmarinic acid	6.6	361.0	163.0	12	11	10
phenolic acid	Sinapic acid	5.4	225.0	175.0	20	19	10
phenolic acid	Syringic acid	4.8	199.0	155.0	20	13	10
phenolic alcohol	Caffeyl alcohol	4.5	165.0	147.0	-20	-16	-7
phenolic alcohol	Coniferyl alcohol	5.2	163.0	131.0	45	13	12
phenolic alcohol	Sinapyl-alcohol	5.1	209.0	194.0	-40	-18	-9
phenolic alcohol	Vanillyl alcohol	4.2	137.0	122.0	20	23	14
phenolic aldehyde	2,5-Dihydroxybenzoic acid	6.8	153.0	108.0	-15	-28	-13
phenolic aldehyde	3,4-Dihydroxybenzaldehyde	4.6	139.0	93.0	50	19	10
phenolic aldehyde	3,4-Dihydroxybenzoic acid	6.2	153.0	108.0	-15	-28	-13
phenolic aldehyde	3,4-Dimethoxycinnamic acid	4.1	209.1	163.0	21	27	16
phenolic aldehyde	3,5-Dihydroxybenzaldehyde	6.2	139.0	111.0	50	15	12
phenolic aldehyde	3,5-Dihydroxybenzoic acid	4.1	153.0	109.0	-15	-18	-13
phenolic aldehyde	3,5-Dimethoxybenzaldehyde	7.9	167.0	139.0	20	15	14
phenolic aldehyde	3,5-Dimethoxybenzoic acid	6.9	183.0	124.0	25	21	14
phenolic aldehyde	4-Hydroxybenzaldehyde	5.3	121.0	92.0	-20	-28	-11
phenolic aldehyde	5-Hydroxyconiferaldehyde	5.2	193.0	178.0	-30	-20	-9
phenolic aldehyde	5-Hydroxyconiferyl-alcohol	5.5	195.0	180.0	-135	-18	-9
phenolic aldehyde	Caffeyl aldehyde	5.3	163.0	135.0	-40	-24	-13
phenolic aldehyde	Coniferaldehyde	6.1	179.0	147.0	30	17	10
phenolic aldehyde	Sinapaldehyde	5.1	209.0	177.0	30	15	10
phenolic aldehyde	Vanillin	5.5	153.0	92.9	50	19	10
phenylpropanoid	5-Hydroxy-ferulic acid	4.8	209.0	150.0	-40	-24	-15
phenylpropanoid	Biochanin A	6.3	283.0	267.9	-120	-30	-13
polyketide-derived mycotoxin	Citrinin	12	251.0	233.0	101	23	12
polyphenol	Ellagic acid	5.1	301.1	284.0	-150	-40	-13
pryridine alkaloid	Trigonelline	1.0	138.0	92.0	170	29	10
pyridoisoquinoline	Emetine	5.5	481.0	246.0	180	47	12
pyrrolizine alkaloid	Erucifoline	2.9	350.2	120.0	125	37	14
sesquiterpene	Abscisic acid	6.4	263.0	153.0	-40	-16	-7
sesquiterpene	Alpha-Cyperone	11.1	219.0	111.0	109	29	12
sesquiterpene	Artemisinin	9.4	283.0	265.1	13	11	12
sesquiterpene lactone	Heptelidic Acid	7.6	279.0	205.0	-33	-12	-11
stilbenoid	Piceid	5.1	389.0	227.0	-90	-20	-11
stilbenoid	Pinosylvin	7.9	213.0	135.0	50	19	14
stilbenoid	Pterostilbene	8.9	255.0	240.0	-80	-26	-13
stilbenoid	Resveratrol	6.3	229.0	135.0	50	19	14
stilbenoid	t-trimethoxyresveratrol	10.4	271.0	152.0	50	73	16
stilbenol	3-Hydroxystilbene	9.2	197.0	119.0	45	17	14
terpenoid indole alkaloid	7-Hydroxymitragynine	4.0	415.0	190.0	60	37	10
trihydroxybenzoic acid	Gallic acid	3.3	169.0	125.0	-30	-20	-13
triterpenoid	Enoxolone	11.0	471.0	189.0	230	45	10

The compounds were detected and quantify using an Agilent 1290 Infinity II liquid chromatography system coupled to a hybrid Triple Quadrupole 6500+ from ABSciex (Framingham, MA). The extracts were kept at 10°C in an auto-sampler, and the phytochemicals were separated at 30°C using a reverse phase C18 Symmetry column (4.6 x 75 mm; 3.5 µm) coupled to a Symmetry C18 pre-column (3.9 x 20 mm; 5 µm) from Waters (Milford, MA). The liquid chromatography gradient was made of 0.1% (v/v) acetic acid in acetonitrile (A) and 0.1% (v/v) acetic acid in water (B). The total LC-MS/MS run was 15 min with a flow rate of 800 µL/min. The following gradient was applied to resolve the polyphenols: B= 0-1.0 minute 98%, 1.0-7.0 min 42%, 7.0-9.0 min 20%, 9.0-11.0 min 10%, 11.0-13.0 min 10%, 13.0-13.1 min 98%, and 13.1-15.0 min 98%. The injection needle was rinsed with 50% aqueous methanol. Five µL of external standard mixtures and 5 µL of biological sample were injected onto the column.

Electrospray ionization with polarity switch was applied to the extracts to acquire mass spectra of the different analytes. The settling time between each polarity was 15 msec. Phytochemicals were simultaneously detected as precursor ion/product ion pair using multiple reaction monitoring (MRM) at first to record the retention time for each of the polyphenols considered in this study (see **Table 1**). The retention times for the compounds were reported in the LC-MS/MS method to create scheduled MRM with MRM detection windows of 60 sec. The cycling time was set to 1.1 sec and the dwell time varied depending on the number of MRMs triggered at a specific point of time during the LC-MS/MS acquisition. The dwell time ranged from 3 to 250 msec. The source parameters for both modes were identical, and they were as followed: 4,500 V for the ionspray voltage, 40 V for the curtain gas, 550°C for the temperature, 50 psi for the nebulizer gas (GS1), 60 psi for the heating gas (GS2), and “Medium” for the collision activated dissociation (CAD).

#### 2.6.2 Data acquisition and processing

Analyst 1.7 software (AB Sciex, Framingham, MA) was used to acquire the data whereas MultiQuant v3.0.3 (AB Sciex, Framingham, MA) was used to integrate the peaks corresponding to the different phytochemicals. Metabolite quantification was performed as previously explained (Arias et al., 2022). Briefly, the total amount of each analyte was calculated using the trans-cinnamic acid-β,2,3,4,5,6-d6 internal standard area, and the known concentration of its corresponding external standard run in parallel to the samples.

#### 2.6.3 Standard curves: Limit of detection, limit of quantification, and linearity range

To determine the limits of detection (LOD) and quantification (LOQ), and the linearity range, standard curves were generated for each metabolite as previously described (Cocuron et al., 2014; Cocuron et al., 2019) with at least six points. Each standard curve was performed in five replicates.

#### 2.6.4 Recovery efficiency, matrix effect, and accuracy intra- and inter- assay

Recovery efficiency (RE) and accuracy intra- and inter- assay were determined using five coffee leaf pseudoreplicates as previously described (Cocuron et al., 2019). To assess the matrix effect (ME), five coffee leaf pseudoreplicates were used according to the procedure previously published (Cocuron et al., 2017) and the following equation:


$$ME=\left(\frac{Analyte\;peak\;area_{Sample\;spiked\;after\;extraction}-\;Analyte\;peak\;area_{Sample}}{Average\;analyte\;peak\;area_{External\;standard}}\times 100\%\right)-100\%$$

In these conditions, a negative value indicates an ion suppression whereas a positive value depicts an ion enhancement due to ME.

### 2.7 Statistical analyses

Principal component analysis (PCA), partial-least square discriminant analysis (PLS-DA), heatmap and ANOVA-simultaneous component analysis (ASCA), for both untargeted and targeted analyses, were performed after log-transformation and auto-scaling, using MetaboAnalyst 5.0 (Chong et al., 2018).

## 3 Results

### 3.1 Untargeted metabolomics

First, the extraction procedure was optimized using a standard mixture containing alkaloids, cinnamate esters, and flavonoids. For that purpose, different solvents/additive and multiple sonication time/temperature combinations were tested: i) defatting beforehand of after extraction with hexanes; ii) methyl ter-butyl ether, ethyl acetate, different percentages of methanol/water as solvents; iii) acetic acid (1%) as additive; iv) under sonication for 10 to 30 min, at temperatures varying from 25 to 40 °C (data not shown). The extracts were injected in the LC-HR-Q-TOF using the same column and solvents described in Cocuron et al. (2019) but a different gradient (see Materials and Methods section). The method which resulted in the best recovery for the most diverse set of metabolites was using two rounds of extraction: the first one 100% methanol, and the second one methanol/water (30:70, v/v) with sonication rounds of 20 min each at 35-40 °C. This procedure was adopted to extract phytochemicals from coffee and wild *Rubiaceae* leaves.

Leaves from three different *Rubiaceae* species were collected in Costa Rica: i) one commercial coffee (CC), *Coffea arabica* cv. Obatã IAC 1669-20 from a plantation, and ii) two wild *Rubiaceae*, *Isertia hankeana* (WR1) and *Simira maxonii* (WR2) from the rainforest. Leaves were freeze-dried and reduced to powder. Secondary metabolites were extracted from leaf powder using the optimized extraction procedure described above, adding a filtering step , and analyzed *via* LC-HR-Q-TOF in positive and negative modes.

**Figure 1:**
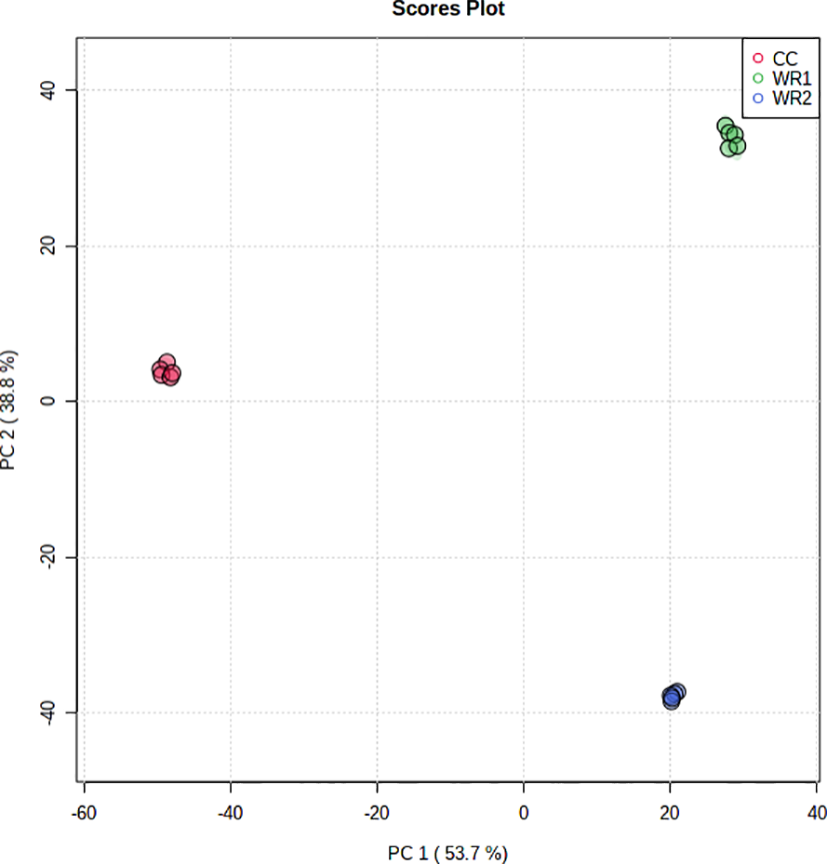
PCA analysis performed on features from untargeted metabolomic analysis in the positive mode of leaf extracts of commercial coffee (CC) and two wild *Rubiaceae* species (WR1 and WR2) collected in Costa Rica. The extractions and analyses were performed with five pseudoreplicates per species.

Runs in the positive and negative mode resulted in 19,000 and 24,000 features, respectively. Principal component analysis (PCA) of unprocessed data (wiff files) from the positive mode, obtained by the untargeted analysis of the leaf extracts, resulted in three widely separated clusters, grouping the five pseudoreplicates from each plant species together (**Figure 1**). The principal component (PC 1) explained 53.7% of the variance, separating the CC from WR1 and WR2, whereas the PC 2 explained 38.8% of the variance, separating all species. This can be interpreted as those three plant species having significantly different metabolic profiles.

After chromatogram alignment, data curation was performed on peak intensities, excluding features with signal lower than one million counts per second and not present on all five pseudoreplicates. Features from positive and negative modes were merged, and when they were present on both modes, only the ones with the highest area were selected. This resulted in 324 features in total within the three species extracts and their pseudoreplicates (**Supplemental Table 1**). National Institute of Standards and Technology (NIST17, Gaithersburg, MD) and in-house library identified 69 and 81 metabolites in the negative mode and the positive mode, respectively. As previously mentioned, for phytochemicals detected in both modes, the highest area was reported (**Table 2**). Thirty-one families of phytochemicals were identified: flavone, flavone C-glycoside, flavonol, indole alkaloid, monolignol phenylpropanoid and triterpenoid were the most common families of compounds found in leaf extracts (**Table 2**). Interestingly, flavones and terpenes were among the families of phytochemicals that were not present in CC but were detected in the wild *Rubiaceae* leaves (**Table 2**). For instance, 6,2’-dihydroxyflavone, afzelin and apigenin were the flavones not detected in CC leaves extracts. Also, robinin, bisdemethoxycurcumin, and madecassic acid were only detected in the wild *Rubiaceae* leaves WR1 and WR2, while epicatechin, vicenin 2, isoquercetrin, 2,3-dehydrosilybin, theobromine, theophylline, trans-3-coumaric acid, and neomangiferin were only present in CC (**Table 2**).

**Table 2:** Families of phytochemical detected by untargeted metabolomics.

Family	Spectral library match	mz/RT (+/-)	CC	WR1	WR2
acyl glycine	Hippuric acid	180.0596/2.1 (-)	3.31E+05 ± 1.24E+04	2.27E+05 ± 2.45E+04	2.06E+05 ± 4.25E+03
amine	Tyramine	138.0913/0.9 (+)	3.37E+06 ± 1.35E+05	5.53E+04 ± 3.27E+03	u.d
benzopyrone	Coumarin	147.0439/10.6 (+)	2.15E+04 ± 3.32E+03	5.58E+05 ± 2.78E+04	5.12E+05 ± 2.93E+04
cinnamate ester	Neochlorogenic acid	353.0877/6.7 (-)	1.08E+07 ± 2.04E+06	1.46E+06 ± 3.03E+05	3.58E+05 ± 7.17E+04
dihyroflavonol	Dihydrokaempferol	287.0563/9.7 (-)	8.64E+04 ± 4.66E+03	1.19E+05 ± 4.98E+03	1.90E+04 ± 1.52E+03
diterpenoid	Gibberellic acid	345.1345/8.4 (-)	u.d	u.d	1.30E+06 ± 9.65E+05
flavan 3-ol	Catechin	289.0715/6.7 (-)	3.99E+05 ± 5.85E+04	1.85E+04 ± 3.20E+03	4.03E+05 ± 1.34E+04
flavan 3-ols	Epicatechin	289.0715/7.0 (-)	1.36E+05 ± 1.57E+04	u.d	u.d
flavanone	Eriodictyol	287.0561/6.8 (-)	9.14E+04 ± 7.19E+03	u.d	2.01E+05 ± 4.65E+03
flavone	6,2’-Dihydroxyflavanone	257.0675/8.3 (-)	u.d	2.16E+06 ± 1.15E+05	9.32E+03 ± 9.54E+02
flavone	Afzelin	431.0978/9.7 (-)	u.d	6.86E+04 ± 7.93E+03	4.07E+04 ± 5.71E+03
flavone	Apigenin	269.0458/11.9 (-)	u.d	u.d	2.69E+05 ± 1.64E+04
flavone	Apigenin 7-glucoside	431.0976/8.8 (-)	2.21E+04 ± 3.10E+03	6.12E+04 ± 4.01E+03	5.06E+03 ± 1.76E+03
flavone	Luteolin	287.0550/8.4 (+)	6.95E+04 ± 9.28E+03	7.96E+05 ± 4.60E+04	2.63E+06 ± 1.19E+05
flavone	Naringenin	273.0755/9.7 (+)	9.78E+04 ± 4.34E+03	4.01E+04 ± 1.16E+04	u.d
flavone C-glycoside	Kaempferol-3-O-rutinoside	593.1525/8.0 (-)	4.36E+04 ± 6.57E+03	3.33E+06 ± 1.38E+05	4.34E+06 ± 2.98E+05
flavone C-glycoside	Kaempferol-7-O-Neohesperidoside	595.1673/8.0 (+)	3.70E+04 ± 5.42E+03	1.98E+06 ± 6.99E+04	3.19E+06 ± 2.32E+05
flavone C-glycoside	Quercetin 3-glucoside	463.0881/8.0 (-)	4.48E+06 ± 3.81E+05	8.91E+04 ± 1.24E+04	1.41E+05 ± 1.16E+04
flavone C-glycoside	Rutin	611.1617/7.7 (+)	1.94E+06 ± 1.45E+05	5.90E+04 ± 5.04E+03	1.54E+04 ± 1.53E+03
flavone C-glycoside	Vicenin 2	595.1518/6.5 (-)	1.64E+06 ± 1.33E+05	u.d	u.d
flavonoid	Tiliroside	593.1311/10.4 (-)	u.d	u.d	6.30E+05 ± 4.19E+04
flavonoid glycoside	Isoquercetrin	465.1034/8.0 (+)	2.31E+06 ± 1.65E+05	u.d	u.d
flavonoid glycoside	Nepetin 7-glucoside	479.1190/9.3 (+)	u.d	u.d	2.77E+05 ± 1.64E+04
flavonol	Kaempferol	285.0401/12.3 (+)	2.72E+04 ± 3.74E+03	1.64E+05 ± 1.01E+04	1.28E+05 ± 5.24E+03
flavonol	Quercitrin	447.0947/8.4 (-)	1.40E+05 ± 4.23E+04	1.95E+06 ± 8.08E+04	5.65E+06 ± 2.06E+05
flavonolignan	2,3-Dehydrosilybin	479.0823/8.0 (-)	1.96E+05 ± 2.74E+04	u.d	u.d
glycosyloxyflavone	Robinin	739.1904/10.2 (-)	u.d	1.18E+06 ± 8.03E+04	5.27E+03 ± 1.06E+03
hydroquinone	3,4-Dihydroxybenzoic acid	153.0196/4.9 (-)	1.05E+06 ± 1.21E+05	2.95E+06 ± 2.65E+05	1.91E+05 ± 7.31E+03
indole alkaloid	Theobromine	181.0715/4.4 (+)	5.73E+06 ± 2.75E+05	u.d	u.d
indole alkaloid	Theophylline	181.0715/5.1 (+)	5.16E+05 ± 2.53E+04	u.d	u.d
indole alkaloid	Yohimbine/Rauwolscine	355.2026/7.1 (+)	1.01E+04 ± 3.91E+03	1.46E+06 ± 6.27E+04	4.89E+04 ± 1.93E+04
indolizine	Isorhyncophylline	385.2123/7.0 (+)	6.02E+04 ± 1.73E+04	3.06E+06 ± 1.15E+05	9.46E+04 ± 3.38E+03
methylxanthine alkaloid	Caffeine	195.0891/6.1 (+)	5.26E+08 ± 1.56E+07	1.50E+05 ± 4.41E+04	1.69E+05 ± 1.89E+04
monohydroxybenzoic acid	Gentisic acid	153.0191/6.0 (-)	1.53E+05 ± 1.10E+04	1.14E+05 ± 6.57E+03	1.03E+05 ± 3.87E+04
monohydroxybenzoic acid	Vanillic acid	167.0344/8.2 (-)	7.58E+04 ± 6.60E+03	2.80E+04 ± 2.22E+03	4.14E+04 ± 4.94E+03
monolignol	Coumaric acid*	163.0403/6.1 (-)	4.51E+06 ± 5.51E+05	1.26E+05 ± 3.99E+03	8.31E+04 ± 2.90E+03
monolignol	Scopoletin	191.0352/8.4 (-)	4.12E+04 ± 4.89E+03	2.81E+05 ± 1.29E+04	2.30E+04 ± 1.40E+03
monolignol	trans-3-Coumaric acid*	147.0440/7.8 (+)	1.07E+06 ± 1.77E+05	u.d	u.d
oxopurine alkaloid	1,3,7-Trimethyluric acid	209.0684/5.3 (-)	5.81E+05 ± 7.30E+04	3.13E+03 ± 7.31E+02	2.72E+03 ± 2.84E+02
oxylipin	Methyl salicylate	153.0544/7.2 (+)	5.84E+05 ± 2.62E+04	5.59E+04 ± 1.96E+04	1.01E+04 ± 9.88E+02
phenolic aldehyde	2,5-Dihydroxybenzaldehyde	137.0247/6.2 (-)	4.28E+06 ± 1.86E+05	5.52E+05 ± 3.22E+04	1.01E+05 ± 1.23E+04
phenylpropanoid	Benzoic acid	121.0309/7.4 (-)	1.87E+06 ± 8.04E+04	5.18E+06 ± 2.20E+05	5.11E+05 ± 2.90E+04
phenylpropanoid	Esculetin	177.0198/6.6 (-)	1.20E+06 ± 4.69E+05	1.31E+05 ± 1.72E+04	7.18E+04 ± 5.87E+03
phenylpropanoid	Esculin	339.0719/6.4 (-)	3.49E+04 ± 1.28E+04	9.44E+04 ± 2.03E+04	5.19E+04 ± 3.85E+03
phenylpropanoid	p-Methoxycinnamic acid	207.1011/8.1 (+)	7.30E+04 ± 6.47E+03	2.94E+04 ± 1.03E+04	u.d
phenylpropanoid	trans-Cinnamic acid	181.0861/10.7 (+)	u.d	6.00E+06 ± 3.18E+05	u.d
polyphenol	Bisdemethoxycurcumin	309.0964/8.9 (+)	u.d	5.08E+05 ± 3.52E+04	1.08E+04 ± 1.64E+03
polyphenol	Caffeic acid	179.0349/6.7 (-)	4.58E+05 ± 2.24E+04	2.08E+05 ± 1.01E+04	1.25E+05 ± 1.69E+04
pyridine alkaloid	Trigonelline	275.1030/1.0 (+)	2.77E+07 ± 8.40E+05	6.01E+04 ± 9.90E+03	7.92E+03 ± 1.06E+03
sesquiterpene	Abscisic acid	263.1286/10.7 (-)	7.83E+04 ± 4.48E+03	2.96E+04 ± 8.94E+02	1.43E+04 ± 5.80E+02
trihydroxyanthraquinone	Chrysophanol	255.0649/17.6 (+)	u.d	u.d	1.66E+05 ± 1.28E+04
trihydroxybenzoic acid	Gallic acid	169.0140/3.5 (-)	2.23E+05 ± 1.88E+04	4.58E+04 ± 9.22E+03	u.d
triterpene	Sumaresinolic acid	473.3626/11.9 (+)	u.d	u.d	4.91E+05 ± 4.77E+04
triterpenoid	Madecassic acid	503.3371/15.2 (-)	u.d	1.42E+05 ± 2.03E+04	1.39E+06 ± 2.18E+05
triterpenoid	Maslinic acid	471.3469/19.8 (+)	5.32E+04 ± 9.97E+03	4.85E+05 ± 4.70E+04	8.16E+05 ± 4.40E+04
triterpenoid	Soyasaponin I	941.4949/15.3 (-)	u.d	1.01E+05 ± 1.39E+04	u.d
xanthone C-glycoside	Neomangiferin	583.1305/5.3 (-)	4.15E+05 ± 3.03E+04	u.d	u.d

The mass to charge ratio value represents the [M+H]^+^ and [M-H]^-^ for the positive and negative modes, respectively, except compounds marked by an asterisk (*) which denotes a loss of water in the source. The areas of secondary metabolites extracted from leaves of commercial coffee (CC) and two wild Rubiaceae species (WR1 and WR2) ± standard deviation (n = 5 pseudoreplicates) are presented. When metabolites were detected in both polarities, only the one with the highest area was reported. Areas labeled as u.d. were under the limit of detection.

For each compound identified using NIST and homemade spectral libraries with > 70% probability, the mass to charge ratio, retention time in min (mz/RT), and mode of detection (positive +, or negative - polarity) are specified.

To validate the findings from the metabolomic profiling, a targeted metabolomic approach was conducted using known quantities of commercially available phytochemical standards.

### 3.2 Targeted metabolomics

#### 3.2.1 Method development

One hundred eighty-four phytochemical standards were individually infused in a triple quadruple to optimize the mass spectrometry parameters (declustering potential - DP, collision energy - CE and collision cell exit potential - CXP) associated with precursor/product ion (Q1/Q3) as shown in **Table 1**. A multiple reaction monitoring (MRM) scan survey was implemented using these parameters. For the liquid chromatography part, the same column, solvents, and additive than the untargeted metabolomics were used. Mixtures of standards were injected into the LC-MS/MS to obtain the retention time for each phytochemical (**Table 1**). It is important to note that standards with the same Q1/Q3 transition were separated by their retention times, except for hesperetin and homoeriodictyol, isovitexin and vitexin, vicenin 3 and schaftoside, morin and tricetin, luteolin-7,3’-Di-O-glucoside and kaempferol-3-O-sophoroside, luteolin-7-O-glucoside and Kaempferol-7-O-glucoside, genistein-7-O-glucuronide and baicalin. For the LC-MS/MS analysis of the 184 phytochemicals, polarity switching (between positive and negative modes) was used within the same run, and MRM windows (aka scheduled MRM) were set up for each compound according to its retention time.

A calibration curve was performed for each standard to determine the limits of quantification, detection, and the linearity range (**Table 3**). The coefficients of correlations were all above 0.98. Acacetin-7-O-rutinoside was the compound with the lowest limits of quantification and detection, at 0.11 and 0.03 fmol, respectively. On the other hand, 2-hydroxyconiferaldehyde was the compound with highest limits of quantification and detection, at 1724.63 and 517.39 fmol, respectively. Tricetin, 2,5-dihydroxybenzoic acid and 4,5-di-O-caffeoylquinic acid were the compounds with the widest range of quantification, from 32.77 to 1,250,000.00 fmol, 81.92 to 1,250,000.00 fmol and 163.84 to 2,500,000.00 fmol, respectively (**Table 3**).

**Table 3:** Range, linearity coefficient (R^2^), limit of quantification (LOQ) and limit of detection (LOD) of secondary metabolites analyzed in this study.

Metabolite	Range (fmol)	R^2^	LOQ (fmol)	LOD (fmol)
1,3,7-Trimethyluric acid	32.77 – 125,000.00	0.9985	4.54	1.36
2,5-Dihydroxybenzoic acid	81.92 – 1250,000.00	0.9995	52.85	15.86
3,4-Dihydroxybenzaldehyde	131.07 – 32,000.00	0.9941	46.81	14.04
3,4-Dihydroxybenzoic acid	327.68 – 32,000.00	0.9935	62.30	18.69
3,4-Dimethoxycinnamic acid	65.54 – 16,000.00	0.9989	22.91	6.87
3,4-di-O-Caffeoylquinic acid	819.20 – 80,000.00	0.9951	364.09	109.23
3,5-Dihydroxybenzaldehyde	327.68 – 1,250,000.00	0.9983	117.87	35.36
3,5-Dimethoxybenzaldehyde	32.77 – 20,000.00	0.9977	9.55	2.87
3,5-Dimethoxybenzoic acid	13.11 – 8,000.00	0.9923	4.68	1.40
3-Caffeoylquinic acid	163.84 – 40,000.00	0.9965	81.92	24.58
3-Deoxyrobinetin	65.54 – 100,000.00	0.9945	26.43	7.93
3-Hydroxystilbene	327.68 – 200,000.00	0.9852	106.74	32.02
4,5-di-O-Caffeoylquinic acid	163.84 – 2,500,000.00	0.9937	148.95	44.68
4-Ccaffeoylquinic acid	163.84 – 100,000.00	0.9988	68.99	20.70
4-Hydroxybenzaldehyde	16.38 – 10,000.00	0.9939	5.43	1.63
4-Hydroxybenzoic acid	32.77 – 8,000.00	0.9994	10.05	3.02
5-Caffeoylquinic acid	65.54 – 250,000.00	0.9928	40.96	12.29
5-Hydroxyconiferaldehyde	4,096.00 – 2,500,000.00	1.0000	1,724.63	517.39
5-Hydroxydimetyltryptamine	131.07 – 5,120.00	0.9920	46.32	13.89
5-Hydroxytryptophan	16.36 – 62,425.00	0.9936	5.20	1.56
6-Methylcoumarin	16.38 – 4,000.00	0.9990	7.99	2.40
7,8-Dihydroxy-4-methylcoumarin	327.68 – 500,000.00	0.9948	114.98	34.49
7-Hydroxymitragynine	163.84 – 100,000.00	0.9970	59.15	17.74
Abscisic acid	16.38 – 10,000.00	0.9978	3.60	1.08
Acacetin-7-O-rutinoside	0.33 – 500.00	0.9957	0.11	0.03
Afzelin	1.31 – 2,000.00	0.9994	0.31	0.09
alpha-Cyperone	16.38 – 4,000.00	0.9979	2.42	0.72
Anthranilic acid	131.07 – 200,000.00	0.9967	80.41	24.12
Apigenin	32.77 – 8,000.00	0.9955	20.61	6.18
Apigenin-7-glucuronide	1.31 – 5,000.00	0.9997	0.45	0.13
Apigenin-7-O-glucoside	3.17 – 1,936.00	0.9971	1.21	0.36
Apigeninidin	64.93 – 39,628.00	0.9984	13.09	3.93
Arbutin	327.00 – 80,000.00	0.9957	52.10	15.63
Arctigenin	327.29 – 79,905.60	0.9928	87.51	26.25
Artemisinin	13.11 – 8,000.00	0.9966	7.62	2.29
Baicalein	3.28 – 5,000.00	0.9993	2.05	0.61
Baicalin	32.77 – 20,000.00	0.9971	14.83	4.45
Biochanin A	32.77 – 20,000.00	0.9957	20.10	6.03
Brefeldin A	131.07 – 80,000.00	0.9901	42.42	12.73
Caffeic acid	131.07 – 500,000.00	0.9983	48.55	14.56
Caffeine	3.28 – 800.00	0.9984	1.12	0.34
Caffeoyl-shikimate	163.84 – 250,000.00	0.9983	85.11	25.53
Caffeyl alcohol	16.38 – 25,000.00	1.0000	10.40	3.12
Caffeyl aldehyde	6.55 – 25,000.00	0.9961	6.43	1.93
Calycosin	6.55 – 4,000.00	0.9936	1.59	0.48
Catechin	16.38 – 10,000.00	0.9928	2.43	0.73
Chrysin	16.38 – 10,000.00	0.9976	5.67	1.70
Chrysoeriol	3.28 – 800.00	0.9968	1.34	0.40
Cinnamic acid	32.77 – 8,000.00	0.9992	14.63	4.39
Coniferaldehyde	16.38 – 10,000.00	0.9902	4.88	1.46
Coniferyl alcohol	65.54 – 16,000.00	0.9975	24.82	7.45
Corynanthine	1.31 – 320.00	0.9971	0.33	0.10
Coumaric acid	16.38 – 10,000.00	0.9984	7.95	2.39
Cynarin	1,310.72 – 2,000,000.00	0.9910	468.11	140.43
Dihydrokaempferol	13.11 – 3,200.00	0.9902	3.03	0.91
Dihydroquercetin	32.77 – 125,000.00	0.9935	26.21	7.86
Diosmetin	1.31 – 800.00	0.9935	0.56	0.17
Enoxolone	65.54 – 16,000.00	0.9945	10.39	3.12
Epicatechin	3.28 – 5,000.00	0.9952	1.38	0.41
Epigallocatechin	65.54 – 40,000.00	0.9955	11.13	3.34
Eriodictyol	6.55 – 4,000.00	0.9886	1.20	0.36
Erucifoline	16.38 – 25,000.00	0.9970	7.99	2.40
Eupatorin	3.28 – 800.00	0.9930	0.99	0.30
Ferulic acid	65.54 – 16,000.00	0.9914	13.43	4.03
Fisetin	65.54 – 250,000.00	0.9982	20.23	6.07
Flavopiridol	1.31 – 2,000.00	0.9964	0.58	0.17
Gallic acid	163.84 – 625,000.00	0.9944	43.57	13.07
Gallocatechin	65.54 – 40,000.00	0.9957	12.85	3.86
Genistein-7-O-glucuronide	3.28 – 5,000.00	0.9936	1.30	0.39
Genkwanin	6.55 – 4,000.00	0.9933	2.72	0.82
Gibberellic acid	32.77 – 20,000.00	0.9960	11.22	3.37
Gingerol	1,310.72 – 32,000.00	0.9934	379.92	113.98
Ginkgolide A	16.38 – 4,000.00	0.9978	3.19	0.96
Glycitein	6.55 – 640.00	0.9953	2.32	0.70
Glycitin	64.49 – 15,744.00	0.9869	15.10	4.53
Harmane	1.31 – 800.00	0.9928	0.44	0.13
Hesperetin	3.28 – 2,000.00	0.9975	1.22	0.37
Homoeriodictyol	3.28 – 800.00	0.9998	1.21	0.36
Hordenine	3.28 – 2,000.00	0.9995	0.83	0.25
Idaein	163.84 – 250,000.00	0.9999	159.84	47.95
Ipriflavone	1.31 – 128.00	0.9989	0.29	0.09
Isoorientin	32.77 – 20,000.00	0.9991	12.90	3.87
Isophorone	6.47 – 632.32	0.9812	1.32	0.39
Isoquercetrin	6.55 – 4,000.00	0.9979	2.16	0.65
Isorhamnetin	6.55 – 1,600.00	0.9969	1.50	0.45
Isorhamnetin-3-O-glucoside	1.13 – 5,000.00	0.9964	0.81	0.24
Isosakuranetin	3.28 – 2,000.00	0.9923	1.21	0.36
Isoschaftoside	1.31 – 5,000.00	0.9921	0.52	0.16
Isovitexin	1.26 – 4,800.00	0.9969	0.92	0.28
Jasmonic acid	32.77 – 20,000.00	0.9898	6.39	1.92
Kaempferide	6.55 – 10,000.00	0.9924	2.31	0.69
Kaempferitrin	6.55 – 1,600.00	0.9989	3.58	1.07
Kaempferol	32.77 – 8,000.00	0.9986	13.43	4.03
Kaempferol-3-O-glucoside	6.55 – 640.00	0.9967	3.01	0.90
Kaempferol-3-O-glucuronide	6.55 – 25,000.00	0.9974	2.29	0.69
Kaempferol-3-O-rutinoside	6.55 – 1,600.00	0.9996	2.40	0.72
Kaempferol-3-O-sophoroside	20.48 – 5,000.00	0.9997	8.40	2.52
Kaempferol-7-O-glucoside	3.28 – 800.00	0.9988	0.78	0.24
Kaempferol-7-O-Neohesperidoside	3.28 – 2,000.00	0.9946	1.27	0.38
Keracyanin	32.77 – 20,000.00	0.9992	7.38	2.21
Luteolin	13.11 – 8,000.00	0.9983	4.24	1.27
Luteolin-7,3’-Di-O-glucoside	3.28 – 128.00	0.9996	1.84	0.55
Luteolin-7-O-glucoside	1.31 – 2,000.00	0.9927	0.67	0.20
Luteolin-7-O-glucuronide	3.28 – 2,000.00	0.9923	1.62	0.49
Matairesinol	131.07 – 32,000.00	0.9939	40.96	12.29
Mellein	13.11 – 3,200.00	0.9996	3.96	1.19
Methyl-jasmonate	0.33 – 12.80	0.9878	0.12	0.04
Miquelianin	16.38 – 25,000.00	0.9985	9.36	2.81
Mitragynine	6.55 – 25,000.00	0.9977	4.12	1.24
Morin	32.77 – 20,000.00	0.9963	12.90	3.87
Myricetin	327.68 – 1,250,000.00	0.9900	76.20	22.86
Myricetin-3-O-Rhamnoside	32.77 – 125,000.00	0.9925	9.13	2.74
N,N-Dimethyltryptamine	0.63 – 2,389.50	0.9925	0.19	0.06
Naringenin	6.55 – 4,000.00	0.9950	2.86	0.86
Naringenin-7-O-glucoside	1.31 – 800.00	0.9984	0.30	0.09
Naringin	1.31 – 2,000.00	0.9994	0.78	0.23
Neobavaisoflavone	1.31 – 320.00	0.9983	0.33	0.10
Neodiosmin	1.31 – 2,000.00	0.9999	0.46	0.14
Orientin	16.38 – 10,000.00	0.9866	6.94	2.08
Paynantheine	13.11 – 1,280.00	0.9915	6.43	1.93
p-Coumaraldehyde	131.07 – 12,800.00	0.9928	28.81	8.64
p-Coumaryl-alcohol	131.07 – 32,000.00	0.9915	37.24	11.17
Phloretin	3.28 – 2,000.00	0.9939	1.10	0.33
Piceid	32.77 – 20,000.00	0.9936	7.67	2.30
Pinosylvin	32.77 – 20,000.00	0.9995	14.50	4.35
Prunetin	6.55 – 4,000.00	0.9954	5.46	1.64
Psilocybin	131.07 – 80,000.00	0.9982	43.84	13.15
Pterostilbene	13.11 – 8,000.00	0.9922	3.34	1.00
Quercetin	32.77 – 8,000.00	0.9903	7.20	2.16
Quercetin-3,4’-O-diglucoside	3.28 – 800.00	0.9955	0.74	0.22
Quercetin-3-O-galactoside	16.38 – 10,000.00	0.9921	5.57	1.67
Quercitrin	16.38 – 10,000.00	0.9975	9.93	2.98
Rauwolscine	1.31 – 320.00	0.9954	0.32	0.09
Reserpine	3.28 – 5,000.00	0.9938	1.21	0.36
Resveratrol	65.54 – 40,000.00	0.9935	18.41	5.52
Rhamnazin	6.55 – 4,000.00	0.9988	2.32	0.70
Rhamnetin	13.11 – 8,000.00	0.9971	3.05	0.91
Roridin L2	16.26 – 9,926.40	0.9937	7.49	2.25
Rutin	13.11 – 8,000.00	0.9912	3.23	0.97
Sakuranetin	3.28 – 2,000.00	0.9960	1.17	0.35
Salicylic acid	16.38 – 10,000.00	0.9939	4.45	1.34
Schaftoside	3.28 – 5,000.00	0.9950	1.70	0.51
Scopoletin	65.54 – 250,000.00	0.9953	29.93	8.98
Scutellarein	163.84 – 16,000.00	0.9984	77.10	23.13
Seneciphylline	6.55 – 4,000.00	0.9950	2.23	0.67
Sinapaldehyde	32.77 – 8,000.00	0.9975	18.94	5.68
Sinapic acid	131.07 – 32,000.00	0.9949	67.56	20.27
Sinapyl-alcohol	1,310.00 – 2,000,000.00	0.9939	471.48	141.44
Sophoricoside	3.28 – 2,000.00	0.9958	1.20	0.36
Spiraeoside	3.28 – 5,000.00	0.9962	1.86	0.56
Strychnine	16.38 – 10,000.00	0.9974	4.38	1.31
Swertiajaponin	13.11 – 8,000.00	0.9978	4.52	1.36
Swertisin	6.55 – 1,600.00	0.9973	3.58	1.07
Syringic acid	65.54 – 40,000.00	0.9979	30.06	9.02
Tamarixetin	6.55 – 4,000.00	0.9936	1.46	0.44
Theobromine	6.55 – 4,000.00	0.9990	2.32	0.69
Theophylline	3.28 – 2,000.00	0.9992	0.84	0.25
Tomatidine hydrochloride	6.55 – 4,000.00	0.9906	2.13	0.64
Tricetin	32.77 – 1,250,000.00	0.9994	8.38	2.51
Tricin	1.31 – 2,000.00	0.9972	0.58	0.17
Trigonelline	1.31 – 2,000.00	0.9810	0.42	0.12
Tryptamine	16.38 – 4,000.00	0.9987	5.73	1.72
t-Trimethoxyresveratrol	16.38 – 4,000.00	0.9962	4.72	1.42
Tyramine	13.11 – 3,200.00	0.9997	5.24	1.57
Vanillic acid	327.68 – 32,000.00	0.9942	109.23	32.77
Vanillin	16.38 – 10,000.00	0.9943	4.89	1.47
Vanillyl-alcohol	32.77 – 8,000.00	0.9992	10.30	3.09
Vicenin 3	3.28 – 5,000.00	0.9907	1.45	0.43
Vicenin 2	6.55 – 1,600.00	0.9993	2.02	0.61
Vincosamide	1.31 – 800.00	0.9972	0.38	0.11
Vitexin	1.31 – 2,000.00	0.9948	0.60	0.18
Xanthohumol	3.28 – 800.00	0.9971	0.89	0.27
Yohimbine	1.31 – 320.00	0.9966	0.45	0.14

Recovery efficiency (RE) and matrix effect (ME) were assessed for each metabolite. Ninety percent of the metabolites were recovered with an efficiency of at least 50%. Keracyanin and enoxolone were the compounds with the lowest REs of 23.3 and 28.9%, respectively (**Table 4**). On the other hand, 5-hydroxytryptophan and 3,4-di-O-caffeoylquinic acid had REs over 100 (**Table 4**). In parallel, ion suppression or enhancement was measured (i.e. ME > ± 30%) for less than 30% of the phytochemicals. For instance, there was a strong negative ME for 3,4-di-O-caffeoylquinic acid and naringin, underlying ion suppression from the sample matrix. Similarly, 4,5-dicaffeoylquinic acid and gossypetin were the compounds with the highest positive ME, indicating ion enhancement (**Table 4**).

**Table 4:** Recovery efficiency (RE) of secondary metabolites and matrix effect (ME) from coffee leaf extracts.

Family	Metabolite	RE	ME
		(%)	(%)
alkaloid	1,3,7-trimethyluric acid	89.90	-24.20
alkaloid	caffeine	76.10	-29.22
alkaloid	corynanthine	56.00	-24.06
alkaloid	erucifoline	61.10	-6.21
alkaloid	harmane	80.30	-23.95
alkaloid	hordenine	72.30	-44.83
alkaloid	mitragynine	46.70	3.28
alkaloid	seneciphylline	61.40	26.53
alkaloid	tomatidine	35.80	-3.46
amino acid derivative	5-hydroxytryptophan	102.70	-44.92
amino acid derivative	tyramine	74.80	-44.75
anthocyanidin	apigeninidin	66.70	13.19
anthocyanin glycoside	keracyanin	23.30	-7.01
cinnamate ester	3,4-di-O-caffeoylquinic acid	194.30	-182.44
cinnamate ester	4,5-dicaffeoylquinic acid	57.20	122.65
cinnamate ester	4-caffeoylquinic acid	82.20	75.55
cinnamic acid derivative	3,4-dimethoxycinnamic acid	76.50	-25.81
coumarin derivative	6-methylcoumarin	78.10	-24.91
coumarin derivative	7,8-dihydroxy-4-methylcoumarin	79.50	-19.74
coumarin derivative	esculetin	82.60	-3.48
coumarin derivative	mellein	81.20	-20.56
coumarin derivative	scopoletin	68.70	-24.87
dihydroxybenzoic acid	2,5-dihydroxybenzoic acid	77.10	-15.47
dihydroxybenzoic acid	3,4-dihydroxybenzoic acid	76.20	0.76
dihydroxybenzoic acid	3,5-dimethoxybenzoic acid	80.50	-24.28
dimethoxybenzene	3,5-dimethoxybenzaldehyde	82.40	-21.11
diterpenoid	gibberellic acid	66.70	-35.04
diterpenoid	ginkgolide A	58.90	-24.42
flavanol	catechin	81.60	-23.50
flavanol	epicatechin	83.10	15.05
flavanol	epigallocatechin	73.60	-85.83
flavanone	eriodictyol	73.10	-29.82
flavanone	hesperetin	80.20	-20.55
flavanone	homoeriodictyol	72.90	-18.14
flavanone	isosakuranetin	80.10	-20.79
flavanone	naringenin	74.30	-22.78
flavanone O-glycoside	naringenin-7-O-glucoside	63.00	-35.42
flavanone-C-glycoside	swertiajaponin	56.20	21.92
flavanone-O-glycoside	naringin	45.80	-90.06
flavanonol	dihydrokaempferol	71.60	-23.12
flavanonol	dihydroquercetin	69.40	-24.51
flavone	3-deoxyrobinetin	60.00	53.64
flavone	apigenin	78.90	-28.58
flavone	apigenin-7-glucuronide	54.20	-16.12
flavone	baicalein	55.20	7.23
flavone	chrysin	87.30	-34.61
flavone	chrysoeriol	78.80	-21.68
flavone	diosmetin	69.80	-16.06
flavone	eupatorin	77.50	-21.60
flavone	flavopiridol	72.20	80.23
flavone	genkwanin	76.90	-22.52
flavone	luteolin	68.80	-12.86
flavone	myricetin	51.00	-18.64
flavone	scutellarein	50.40	51.01
flavone	tectochrysin	67.80	-18.77
flavone	tricetin	53.80	36.58
flavone C-glycoside	isoorientin	53.50	4.16
flavone C-glycoside	swertisin	55.60	-15.14
flavone-C-glycoside	isoschaftoside	66.20	-22.12
flavone-C-glycoside	isovitexin/vitexin	58.80	-16.56
flavone-C-glycoside	orientin	52.10	11.89
flavone-C-glycoside	Vicenin 2	42.00	-10.71
flavone-O-glycoside	acacetin-7-O-rutinoside	61.80	-9.30
flavone-O-glycoside	apigenin-7-O-glucoside	56.90	-25.08
flavone-O-glycoside	baicalin	62.00	-20.13
flavone-O-glycoside	luteolin-7,3’-di-O-glucoside	64.50	-32.82
flavone-O-glycoside	luteolin-7-O-glucoside	42.40	-27.38
flavone-O-glycoside	luteolin-7-O-glucuronide	69.20	-12.69
flavone-O-glycoside	myricetin-3-O-rhamnoside	44.60	47.77
flavone-O-glycoside	neodiosmin	43.60	-88.65
flavonol	fisetin	72.80	17.56
flavonol	gossypetin	38.30	186.65
flavonol	isorhamnetin	57.70	-13.77
flavonol	kaempferide	73.60	-20.18
flavonol	kaempferol	69.30	-21.47
flavonol	morin	54.00	28.56
flavonol	quercetin	67.10	-17.24
flavonol	quercitrin	58.20	-2.56
flavonol	rhamnazin	60.80	-15.60
flavonol	rhamnetin	61.90	-12.18
flavonol	tamarixetin	66.90	-18.37
flavonol-O-glycoside	afzelin	68.60	-26.06
flavonol-O-glycoside	isoquercetrin	68.40	16.38
flavonol-O-glycoside	isorhamnetin-3-O-glucoside	68.50	2.12
flavonol-O-glycoside	kaempferitrin	52.00	0.79
flavonol-O-glycoside	kaempferol-3-O-glucoside	60.20	28.18
flavonol-O-glycoside	kaempferol-3-O-glucuronide	71.20	-18.36
flavonol-O-glycoside	kaempferol-7-O-glucoside	56.30	-29.40
flavonol-O-glycoside	kaempferol-7-O-neohesperidoside	51.30	2.93
flavonol-O-glycoside	quercetin-3,4’-O-diglucoside	49.20	-21.71
flavonol-O-glycoside	quercetin-3-O-galactoside	53.00	-0.89
flavonol-O-glycoside	rutin	33.80	-48.97
flavonol-O-glycoside	spiraeoside	55.40	-20.78
hydroxybenzoic acid	4-hydroxybenzoic acid	83.00	-36.35
hydroxybenzoic acid	gallic acid	76.40	-19.35
hydroxybenzoic acid	salicylic acid	51.40	-74.19
hydroxybenzoic acid	vanillic acid	82.00	-78.18
hydroxycinnamic acid	caffeic acid	65.30	-17.55
hydroxycinnamic acid	coumaric acid	80.20	-25.62
hydroxycinnamic acid	ferulic acid	75.40	-18.89
indole alkaloid	5-hydroxydimethyltryptamine	91.50	-37.98
indole alkaloid	7-hydroxy mitragynine	59.20	-20.88
indole alkaloid	anthranilic acid	72.60	48.89
indole alkaloid	N,N-dimethyltryptemine	79.30	-0.45
indole alkaloid	psilocybin	82.50	45.11
indole alkaloid	rauwolscine	57.20	-22.77
indole alkaloid	reserpine	30.70	0.24
indole alkaloid	theobromine	76.50	-17.88
indole alkaloid	theophylline	77.00	-25.19
indole alkaloid	tryptamine	72.60	-42.00
isoflavone	biochanin A	74.50	-24.03
isoflavone	calycosin	70.90	-21.78
isoflavone	glycitein	81.40	-22.87
isoflavone	ipriflavone	66.40	-24.26
isoflavone	neobavaisoflavone	65.30	-21.12
isoflavone	prunetin	75.60	-17.48
isoflavone-O-glycoside	genistein-7-O-glucuronide	69.30	-19.96
isoflavone-O-glycoside	glycitin	53.50	-12.45
isoflavone-O-glycosylated	sophoricoside	59.00	-43.91
lactone	brefeldin A	68.30	-33.62
lignan	arctigenin	63.50	-21.16
lignan	matairesinol	64.90	-24.19
monocarboxilic acid	cinnamic acid	80.50	-21.71
mycotoxin	neosolaniol	87.90	36.21
mycotoxin	roridin-L2	36.50	-22.76
oxylipin	jasmonic acid	73.30	-25.55
oxylipin	methyl jasmonate	95.30	-30.97
phenol	gingerol	68.80	-20.27
phenolic acid	benzoic acid	90.80	-36.14
phenolic acid	sinapic acid	69.60	-14.47
phenolic acid	syringic acid	80.10	-55.23
phenolic alcohol	caffeyl alcohol	81.80	-33.98
phenolic alcohol	coniferyl alcohol	77.60	-20.35
phenolic alcohol	sinapyl alcohol	67.90	-17.88
phenolic alcohol	vanillyl alcohol	83.70	-22.66
phenolic aldehyde	3,4-dihydroxycbenzaldehyde	93.40	41.47
phenolic aldehyde	4-hydroxybenzaldehyde	78.50	-70.69
phenolic aldehyde	caffeyl aldehyde	77.70	-49.70
phenolic aldehyde	coniferaldehyde	77.40	-20.36
phenolic aldehyde	sinapaldehyde	70.10	-56.06
phenolic aldehyde	vanillin	79.60	-24.83
sesquiterpene	abscisic acid	60.60	-29.79
sesquiterpene	artemisinin	49.30	-22.97
sesquiterpene	heptelidic acid	68.30	-20.29
sesquiterpene	α-cyperone	70.30	-19.83
stilbenoid	3-hydroxystilbene	78.20	-23.62
stilbenoid	piceid	50.90	-7.63
stilbenoid	pinosylvin	77.80	-18.70
stilbenoid	pterostilbene	66.80	-33.96
stilbenoid	resveratrol	64.10	-32.92
stilbenoid	t-trimethoxyresveratrol	58.60	-19.81
triterpenoid	enoxolone	28.90	-25.84

To verify the method accuracy, at least one representative of each major phytochemical family was added to coffee leaf extracts at three concentrations: 0.25, 0.5, and 1µM. Intra-day and inter-day accuracy percentages were determined by re-injecting the samples on the same day and on three different days, respectively (**Table 5**). Out of the 25 phytochemicals tested, 18 and 22 had intra-day and inter-day accuracies above 75%, respectively.

**Table 5:** Intra and inter-day accuracy percentages for secondary metabolites added at three different concentrations (0.25, 0.5 and 1 µM) to coffee leaf extracts.

		Intra-day assay	Inter-day assay
Metabolite	Concentration (µM)	n = 5	n=15
1,3,7-trimethyluric acid	0.25	11.3	16.0
	0.50	25.2	19.9
	1.00	16.3	15.9
3,4-dimethoxycinnamic acid	0.25	3.0	2.1
	0.50	3.0	2.6
	1.00	1.0	0.6
3-Hydroxystilbene	0.25	27.7	12.2
	0.50	6.1	6.8
	1.00	2.2	2.9
4-Hydroxybenzoic acid	0.25	14.5	17.0
	0.50	15.5	18.5
	1.00	14.0	17.1
5-Hydroxytryptophan	0.25	6.5	4.5
	0.50	46.1	28.7
	1.00	43.6	31.8
Apigeninidin	0.25	7.5	5.4
	0.50	9.5	9.2
	1.00	11.4	11.0
Arctigenin	0.25	7.1	7.7
	0.50	3.4	3.7
	1.00	2.6	3.4
Biochanin A	0.25	6.1	8.5
	0.50	3.6	4.8
	1.00	1.0	1.9
Coniferaldehyde	0.25	7.9	7.0
	0.50	6.4	5.8
	1.00	3.0	3.5
Coniferyl alcohol	0.25	30.8	26.1
	0.50	28.3	23.1
	1.00	18.6	17.1
Cyperone	0.25	7.9	3.2
	0.50	1.8	2.3
	1.00	1.0	1.6
Dihydrokaempferol	0.25	5.9	5.3
	0.50	7.6	7.5
	1.00	6.2	5.7
Epicatechin	0.25	29.3	18.5
	0.50	31.7	23.6
	1.00	4.5	9.2
Ferulic acid	0.25	15.3	11.3
	0.50	17.1	14.6
	1.00	1.6	4.6
Gibberellic acid	0.25	5.5	8.9
	0.50	1.5	7.7
	1.00	4.3	8.2
Genistein-7-O-glucuronide	0.25	3.4	8.2
	0.50	1.8	8.2
	1.00	7.6	7.3
Isorhamnetin-3-O-glucoside	0.25	8.4	5.4
	0.50	4.6	5.3
	1.00	0.5	1.7
Jasmonic acid	0.25	6.5	6.7
	0.50	2.6	4.1
	1.00	2.4	3.0
Luteolin	0.25	1.1	4.9
	0.50	4.0	5.2
	1.00	5.0	3.8
Naringenin	0.25	3.1	3.3
	0.50	0.1	1.6
	1.00	2.5	1.6
Orientin	0.25	8.1	15.1
	0.50	8.5	20.3
	1.00	14.0	17.0
Scopoletin	0.25	4.5	4.9
	0.50	6.7	7.5
	1.00	1.6	3.2
Sinapic acid	0.25	30.1	20.4
	0.50	28.7	24.3
	1.00	4.2	6.5
Swertiajaponin	0.25	0.8	25.7
	0.50	6.0	26.9
	1.00	41.4	40.1
Theobromine	0.25	35.4	17.9
	0.50	16.7	12.5
	1.00	5.2	9.1

Intra-day and inter-day accuracy percentages were determined by re-injecting the samples on the same day (n = 5) and on three different days (n = 15), respectively.

#### 3.2.2 Application of the analytical method to quantify phytochemicals in leaf extracts from commercial coffee and wild *Rubiaceae*

**Figure 2:**
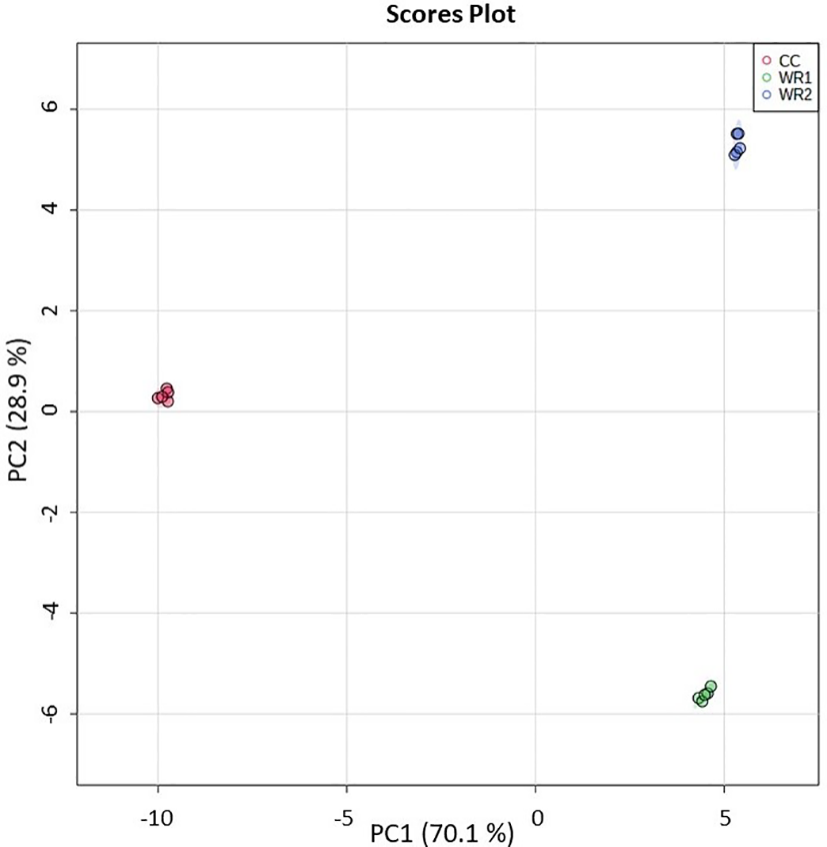
PCA analysis performed on targeted metabolomic analysis data from extracts of commercial coffee (CC) and two wild *Rubiaceae* species (WR1 and WR2). The extractions and analyses were performed with five pseudoreplicates per species. The shaded regions represent the 95% confidence intervals.

Validation of the method was performed using the same biological samples and extracts as the untargeted metabolomics (**Supplemental Table 2**). From the 184 phytochemicals monitored by LC-MS/MS, 74 were quantifiable in at least one of the *Rubiaceae* species. Interestingly, all the 74 phytochemicals were significantly different in at least one comparison by ANOVA (p<0.05). The PCA was consistent with the one from the untargeted metabolomic analysis (**Figure 1**), resulting in three separate clusters (**Figure 2**). The principal component 1 (PC 1) explained 70.1% of the variance, separating the CC from the wild *Rubiacaea*, whereas the PC 2 explained 28.9% of the variance, separating all species.

**Figure 3:**
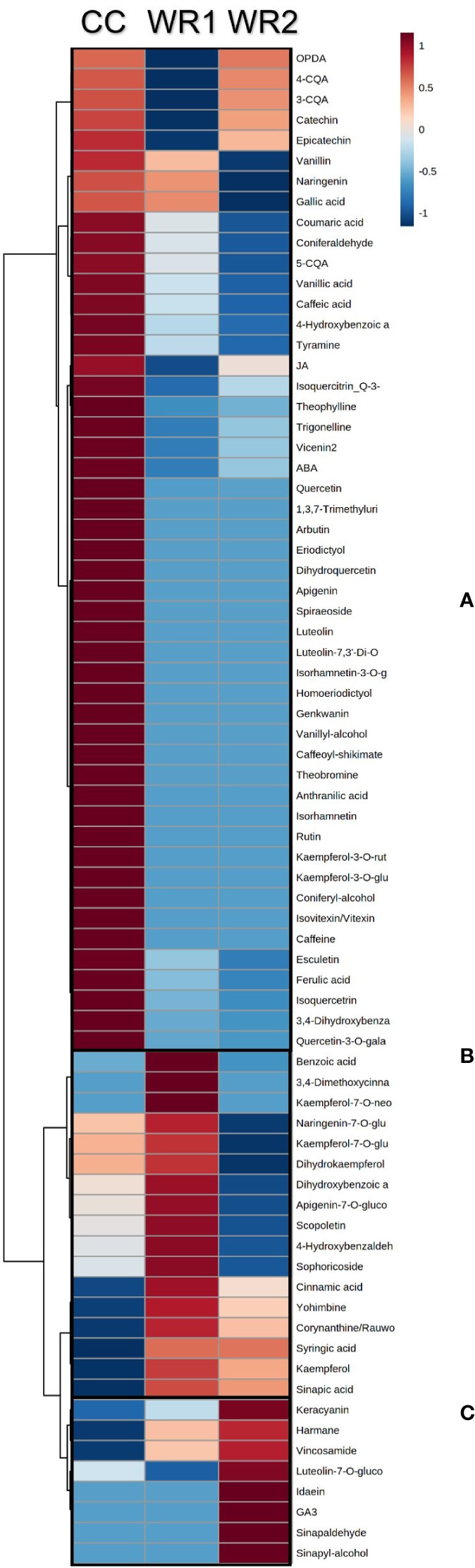
Heatmap analysis of the polyphenols from extracts of coffee (CC) and two wild *Rubiaceae* (WR1 and WR2) detected by targeted metabolomics. The metabolomics data were normalized by log transformation, mean-centered, and divided by the standard deviation of each variable. Ward’s hierarchical clustering algorithm was used to group metabolites that have the same distribution pattern in the heat map. Color scale represents metabolite relative intensity, with the darkest red and blue symbolizing the highest and the lowest values, respectively. Boxes highlight metabolites that are higher in CC **(A)**, in WR1 **(B)**, and WR2 **(C)**.

Targeted metabolomic analysis revealed compounds that were highly concentrated in commercial coffee leaf extracts when compared to wild *Rubiaceae* extracts (**Figure 3**; **Supplemental Table 2**). Caffeoylquinic acids, like 3, 4 and 5-caffeoylquinic acids, caffeine, trigonelline, vincenin 2, theobromine and others were more abundant in CC (**Figure 3**). Also, similarly to what was found in the untargeted analysis, some compounds were more abundant and even sometimes found exclusively in wild *Rubiaceae* leaf extracts. For instance, the levels of benzoic acid, 3,4-dimethylcinnamic acid and kaempferol-7-O-neohesperidoside, were the highest in WR1 in comparison to CC and WR2 (**Figure 3**). Cinnamic acid, yohimbine, corynanthine/rauwolscine, syringic acid, kaempferol were abundant in WR1 but absent in CC (**Figure 3**). Similarly, the levels of keracyanin, luteolin-7-O-glucoside, idaein, gibberellic acid, sinalpadehyde and sinapyl-alcohol were found to be the highest in WR2, while harmane and vincosamide, abundant in WR2, were not detected in CC (**Figure 3**).

**Figure 4:**
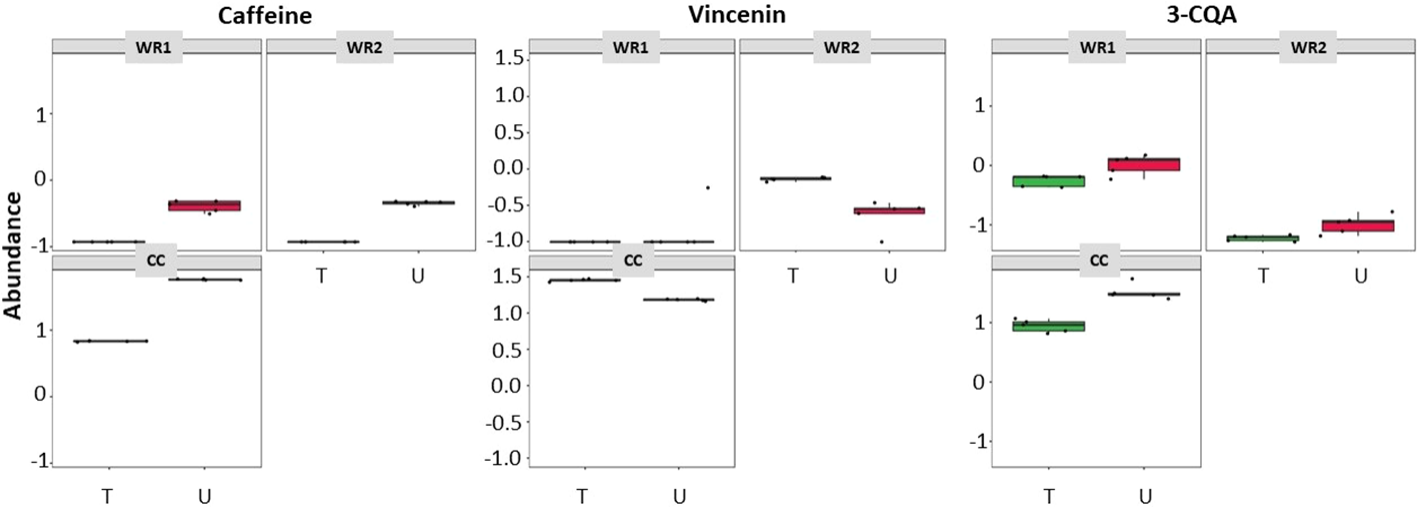
ASCA showing similarities of metabolites detected in coffee and wild *Rubiaceae* leaf extracts detected by targeted (T - green boxes) and untargeted (U – red boxes) metabolomics. 3-CQA is 3-caffeoylquinic acid.

**Figure 5:**
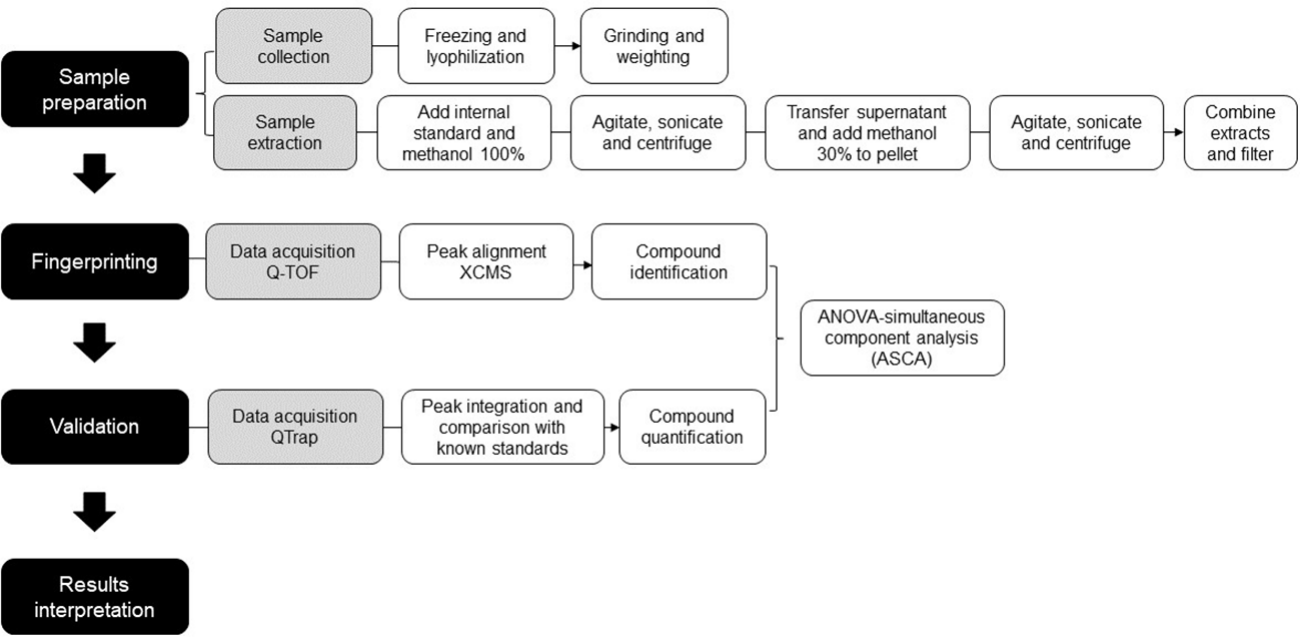
Workflow of the metabolomic platform to identify and quantify polyphenols using liquid chromatography mass spectrometry.

To determine the consistency between untargeted and targeted results, an ANOVA-simultaneous comparative analysis (ASCA) was performed on the 74 polyphenols quantified in our samples, and it revealed that both techniques display similar results. **Figure 4** are three examples of phytochemicals—caffeine, vicenin and 3-caffeoylquinic acid (3-CQA)—that were found to be significantly more abundant in CC using the untargeted (U) metabolomics. These results were validated by a quantitative targeted (T) approach, which confirmed that the three polyphenols were the highest in CC. These examples illustrate the consistency between the untargeted and targeted metabolomics in our pipeline. **Figure 5** depicts the workflow of the metabolomic platform to identify and quantify polyphenols using liquid chromatography mass spectrometry.

## 4 Discussion

Plant secondary metabolites belong to a wide variety of chemical families and are present at very small concentrations, which makes it challenging to recover with a single extraction procedure and to quantify with a sensitive enough analytical technique. Moreover, most phytochemicals are poorly soluble in water, photosensitive and not thermostable, requiring careful procedures for their handling and storage. Choosing the right solvent composition and solid to liquid ratio is critical for isolating plant metabolites (Zhang et al., 2011). In general, the most common solvents are ethanol, methanol, chloroform, and water in different proportions for plant secondary metabolite extraction (Silva et al., 1998; Abubakar and Haque, 2020). Nonetheless, chloroform has a low polarity and is a carcinogen, therefore it is recommended to avoid it (Davidson et al., 2008). In the present study, methanol and water resulted in the most efficient mixture of solvents to recover polyphenols from coffee leaves, combined with sonication at 35-40°C. Indeed, ultrasonication has been widely used to reach higher yields of natural compounds (Annegowda et al., 2010; Masson et al., 2010; Hasan et al., 2017). For instance, ultrasonic-assisted extraction has been used in coffee leaves to improve the extraction of caffeine, trigonelline, rutin, chlorogenic acids, and mangiferin (Chen et al., 2020b). Furthermore, steroidal alkaloids have been successfully recovered from potato peel using ultrasound assisted extraction, obtaining at least 1.5 times more compounds in comparison with other extraction technique (Hossain et al., 2014). A comparative study in *Hibiscus* spp. concluded that using methanol and sonication resulted in better yields of phytosterols (Soares Melecchi et al., 2006). Sonication was also used to successfully extract quinones and flavonoids of six different species of *Dosera* sp. (Marczak et al., 2005). Other extraction techniques cited in literature involve microwave-assisted extraction, pressurized-liquid extraction and supercritical fluid extraction (Zhang et al., 2011; Abubakar and Haque, 2020). However, those are more time consuming and costly techniques (Roopashree and Naik, 2019).

Untargeted metabolomics aims to capture the whole metabolome (Matsuda et al., 2009), while targeted analysis focuses on the use of commercially available standards to detect and measure the quantity of metabolites present in a biological sample (Shimizu et al., 2018). It is therefore essential to have previous knowledge of the sample composition before performing targeted analysis. Most studies perform untargeted or targeted analysis, but usually do not combine both. However, the combination of both approaches, like the present study, creates a powerful tool to differentiate profiles and then detect/quantify the differences highlighted by metabolite fingerprinting, confirming the results. For instance, untargeted and targeted associated research on three *Coffea* sp. could differentiate the species metabolic profiles of leaf and fruit extracts. Additionally, five phytochemicals (caffeine, mangiferin and three caffeoylquinic acids) were identified, corroborating the identity of the differentiated metabolites between species and tissues (Montis et al., 2022). Also, Zhang et al. characterized cyclopeptides in 20 species of *Rubia* sp. (*Rubiaceae*) using LC-MS/MS (Zhang et al., 2018). Similarly to our study, the authors combined untargeted and targeted metabolomics using LC coupled to a triple TOF and a triple quadrupole, respectively, which provides reliability, precision, and sensitivity, with the additional advantage of requiring very small amounts of plant tissue. Nevertheless, our study reports an extraction that can be performed in less than two hours, and was optimized to recover a wide variety of compounds (over 40 families of phytochemicals). Indeed, 90% of the phytochemicals in our study had a recovery higher than 50%. Finally, 184 phytochemicals can be quantified in a sensitive and specific manner within a single LC-MS/MS run of 15min with polarity switch, which is particularly suitable for high-throughput analyses.

Our study compared the leaf phytochemical profiles from one commercial coffee and two wild *Rubiaceae* species. First, the untargeted metabolomics analysis identified 31 families of phytochemicals, of which flavone, flavone C-glycoside, flavonol, indole alkaloid, monolignol phenylpropanoid and triterpenoid were the most common families (**Table 2**). Several flavones and terpenes were not present in CC. Indeed, flavones 6,2’-dihydroxyflavone, afzelin and apigenin, the flavonoid tiliroside, flavonol nepetin-7-glucoside and the glycosylflavone robinin were not detected in CC leaves. Interestingly, flavonoids are the most abundant secondary metabolites in human diet (Alara et al., 2021): afzelin has been reported to have anti-inflammatory action (Kim et al., 2019a) while apigenin is an antioxidant and has anticancer properties (Shankar et al., 2017; Kim et al., 2019b). Flavonoids also play important roles in plant development and response to stress (Du Fall and Solomon, 2011; Nakabayashi and Saito, 2015). For example, robinin has been associated with plant drought resilience, as Chrysanthemum plants previously treated with this flavone had enhanced response to water stress and were able to maintain turgor pressure (Elansary et al., 2020). Gibberellic, sumaresinolic and madecassic acids, and soyasaponin were also absent in CC leaf extracts. Gibberellic acid is a plant hormone with multiple functions in growth regulation, flowering and stress response signaling (Bari and Jones, 2009; Schwechheimer and Willige, 2009; Iftikhar et al., 2019; Nagar et al., 2021). Madecassic acid, on the other hand, has been shown to have some medicinal properties, such as anti-inflammatory effects (Won et al., 2010), anti-colitis (Xu et al., 2017), and potential anti-cancer agent (Zhang et al., 2014; Valdeira et al., 2019). Equivalently, soyasaponins have been associated with health promoting properties, such as anti-inflammatory, anti-microbial, and cardiovascular protective activities (Guang et al., 2014; Lee et al., 2020; Wang et al., 2020).

Then, the targeted metabolomic analysis identified several compounds that were highly concentrated in CC or WR leaves (**Figure 3**; **Supplemental Table 2**). The most abundant compounds in CC leaves were chlorogenic acids (3,4 and 5-caffeoylquinic acids), caffeine, trigonelline, vicenin 2 and theobromine, which is consistent with previous reports (Campa et al., 2012; Funlayo et al., 2017; Chen, 2019; Cangeloni et al., 2022). Chlorogenic acids are well-known compounds for their antimicrobial activities (Sung and Lee, 2010; Su et al., 2014; Martínez et al., 2017). Indeed, they were shown to have deleterious effects on coffee microbial pathogens: a study showed that coffee plants supplied with silicon–a resistance inducer–had higher levels of chlorogenic acids and were therefore more resistant to *Hemilea vastatrix*, the causal agent of rust (Rodrigues et al., 2011). Caffeine content is one of the most important traits for coffee selection, either to bean processing for beverage consumption or for the pharmaceutical industry (Sawynok, 1995; Leroy et al., 2006; Patay et al., 2017; Carvalho et al., 2019). Having a resourceful method for caffeine detection and quantification, along with other desirable traits, would aid coffee breeders and studies on cultivar development. However, additional research is needed to correlate leaf and berry/bean composition in coffee to perform cultivar selection at earlier stages and speed up the breeding process. The content in kaempferol-7-O-neohesperidoside, kaempferol, yohimbine, corynanthine/rauwolscine, 3,4-dimethylcinnamic, cinnamic, benzoic and syringic acids were more abundant in WR1 leaves. A study in Litchi chinensis seeds revealed that kaempferol-7-O-neohesperidoside had a high cytotoxic activity against lung cancer cells (Xu et al., 2011). Similarly, kaempferol has also shown anti-cancer (Chen et al., 2020a; Kluska et al., 2021; Felice et al., 2022), anti-oxidant (Simunkova et al., 2021) and anti-malarial activities (Somsak et al., 2018), confirming more therapeutic uses of flavonoids. Alkaloids like yohimbine have promising clinical applications (Boğa et al., 2019; Saini et al., 2022), like anti-cancer activity (Jabir et al., 2022), and may be used as chemical markers for botanical selection (Osman et al., 2019). Several polyphenols were more abundant in WR2: harmane, vincosamide, keracyanin, idaein, gibberellic acid, luteolin-7-O-glycoside, synapaldehyde, and sinapyl-alcohol. Anthocyanins play important roles not only in plant reproduction, but also in response to abiotic and biotic stresses (Liu et al., 2018). Additionally, keracyanin and idaein were proven to have potential anti-inflammatory and anti-cancer activities (Natarajan et al., 2016; Santamarina et al., 2021). Monolignols, key components for lignin biosynthesis, are crucial element for cell wall protection against stresses (Gallego-Giraldo et al., 2018; Xie et al., 2018). Synapaldehyde increases reactive oxygen species in plants and has anti-fungal activity (Millan et al., 2022). Also, this metabolite was more abundant in sugarcane resistant to the causal agent of ratoon stunting (Castro-Moretti et al., 2021), associating its role to plant defense. Overall, WR leaves had a higher diversity of phytochemicals in comparison to the CC. This may be due to the farmer selection of CC for fruit size, caffeine content, and yield at the detriment of other stress resistance and adaptation traits.

## 5 Conclusions

The present study reports: i) the optimization of an extraction procedure to recover 42 distinct families of phytochemicals from leaves, ii) the development of a robust and sensitive LC-MS/MS method to quantify 184 secondary metabolites, and iii) the complementarity between the untargeted and targeted metabolomics. This approach was applied to characterize the phytochemicals in three different species of *Rubiaceae*, including two wild species and one commercial coffee. The new targeted metabolomics approach was used to validate the identity of 74 compounds highlighted by the untargeted analysis. This work describes a sensitive and thorough pipeline (**Figure 5**) to detect, classify and quantify secondary metabolites in leaves of coffee and other *Rubiaceae*, and can be further applied to other plant organs and species.

## Data availability statement

The original contributions presented in the study are included in the article/**Supplementary Material**. Further inquiries can be directed to the corresponding author.

## Author contributions

FC-M, J-CC, PC, JCS, and APA designed and conceptualized the project and method development; HC-G, EE-L, PC, and OG-F collected the leaves and prepared plant materials; FC-M and J-CC tested the different extraction methods, performed the LC-MS/MS method development, and method validation; FC-M. performed data processing and statistical analyses; FC-M, J-CC, and APA drafted the manuscript; APA, JCS and PC supervised, coordinated the project, and acquired funding for the project. All authors have read and agreed to the published version of the manuscript.

## Supplementary material

Supplementary Figure 1 PCA scores plot showing clustering of quality control samples for untargeted metabolomics in positive mode **(A)** and negative mode **(B)**. QC, quality control; CC, commercial coffee; WR1 and WR2, wild *Rubiaceae* species.
