# Supplementary figures and images for: A metabolomic platform to identify and quantify polyphenols in coffee and related species using liquid chromatography mass spectrometry
Source: Front Plant Sci. 2023 Jan 6;13:1057645. doi: 10.3389/fpls.2022.1057645 (PMC9852862; doi:10.3389/fpls.2022.1057645)

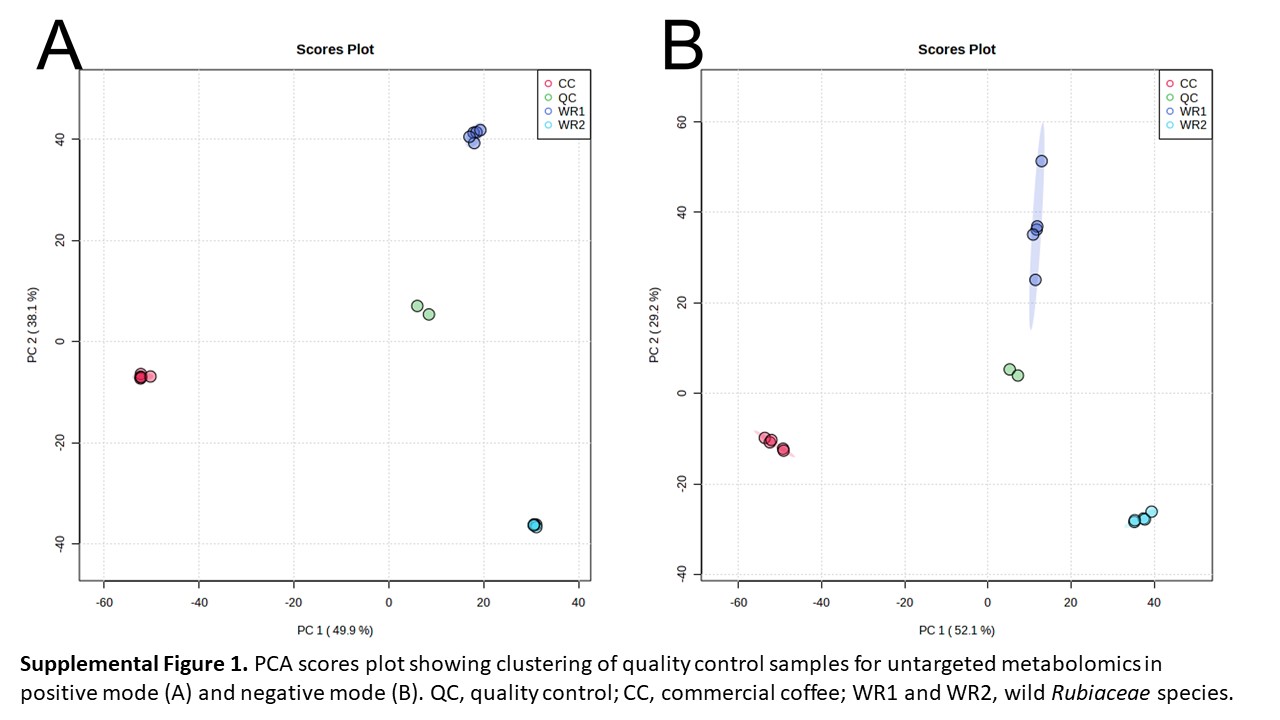

Supplement: Supplementary Figure 1 — PCA scores plot showing clustering of quality control samples for untargeted metabolomics in positive mode (A) and negative mode (B). QC, quality control; CC, commercial coffee; WR1 and WR2, wild Rubiaceae species. [file Image_1.jpeg]
